# Supplementary material for: Performance of tree-building methods using a morphological dataset and a well-supported Hexapoda phylogeny
Source: PeerJ. 2024 Jan 8;12:e16706. doi: 10.7717/peerj.16706 (PMC10782957; doi:10.7717/peerj.16706)
Supplement: Supplemental Information 1 — Operational adaptations were made in the terminal groups, the group-names were updated and edited according to the most recent information and the two matrices were standardized for comparison. [file peerj-12-16706-s001.pdf]

Operational adaptations in Beutel & Gorb (2001) matrix:

Operational adaptations were made in the terminal groups, the group-names were updated and edited according to the most recent information and the two matrices were standardized for comparison.

1) “Chilopoda” and “Symphyla” were removed,
2) “Lepism.-Nicolet.” instead of “Tricholepidion” was chosen as the standard for Zygentoma because it contains less missing data, the monophyly of Zygentoma is strongly supported in most recent studies (Blanke et al., 2014; Song et al., 2016),
3) “Grylloblatodea” was treated as “Xenonomia”, the monophyly of Xenonomia is strongly supported in several studies (Beutel & Gorb, 2006; Terry & Whiting, 2005; Wipfler et al., 2011) and Mantophasmatodea was not known until Klass et al. (2002),
4) “Ensifera”, instead of “Caelifera” was chosen as a standard for Orthoptera because it contains less missing data, the monophyly of Orthoptera is strongly supported in several studies (Beutel & Gorb, 2001; Flook & Rowell, 1997, 1999; Song, 2010, 2015; Zhang et al., 2013),
5) “Heteroptera” was chosen as the standard for Hemiptera, considering that the monophyly of this suborder is less contested than the monophyly of the others suborders, besides that the monophyly of Hemiptera is strongly supported in several studies (Beutel & Gorb, 2001; Cryan & Urban, 2012; Song et al., 2012, 2019). The main reason Orthoptera and Hemiptera are treated as sub-orders in the work Beutel & Gorb (2001) was for the study of the evolution of adhesive pad character within these orders and Hexapoda in general.

Beutel & Gorb (2001) adapted matrix (Nexus format):

#NEXUS
BEGIN DATA;
DIMENSIONS NTAX=32 NCHAR=115;
FORMAT DATATYPE = STANDARD GAP = - MISSING = ? SYMBOLS = "0 1 2 3 4 5 6 7";
MATRIX
Diplura   111000000-00100010000001000000000000100100-------------0??0?00000000(12)(01)0---00000(01)(02)-01000-010000000010100000000000000
Protura   110001000-0012--20100000100000000000001000000-------------0??0?0?000001210---0000301-0?000-110?00000?0000000000000000

Collembola 110001000-00100020000001000001000000100000--------------0??0?

00000111210---0000202000000-1100000000010000000000000

Archaeognatha 10100000100001010000000000000000000000121100--------------0?

000000000000010001000010000000-000100000101000000000000000

Zygentoma 101110111010010101010001010010000000121100--------------0100000000(02)

(01)010110010000100000000-0002010002010000000000000

Ephemeroptera

1011101010100101010100010000100000011411010000000000000000110000000210002100100000

140000000000220000201001000000001000

Odonata

1011101110100101020100010000110000011211010000000000000010110000000(01)210001000000

00100000000010210000201011000000000000

Plecoptera

10111011101001010201000100001100000112110110 1(01)100(01)0000010000100001210002-

00000001000000000(01)022000020101101010000000

Dermaptera 1111101110100101020100010000011000011211011011110200000010(01)

(01)000000121000110000000120000000010220110201010 1(01)001000000

Blattodea

101110111110010102010001000011100101141101101111010000010000000001210001100011001 0

0000000010220000211010010100000000

Isoptera

1011101111100101020100010000111001011411011011000000000010000000001210(01)011000110

0100000000010220000211010110000000000

Mantodea

10111011111001010201000100001110010114110110101101000001000000000121000110001100 10

000000001022000021101000010000000

Embioptera

1111101110110101020100010000111100011211011011000000000010000100001210002-

000000010000000000102201002010101000 1000000

Orthoptera

1011101110100101020100010000011000111311111010110101000100000000001210001100000001 0

000000001022000020101000010000000

Phasmatodea

10111011101001010201000100001111010114110110101101010001000000000121000110000000 1

0000000001022001020101001010000000

Xenonomia 1111101110100101020100010000111 0000-14110---------------?
00000000121000110000000010000000-010?2000020101000010000000

Zoraptera 101110111010010102010001 00?001100?011111011?1100?0000??????0???00?
210102-00000011000???00010?2?0002010101000000000

Thysanoptera 10111011101001013-2101110(01)0001101?011111011011001000?
0010010000001211 10(12)1000000211000000001022000020111002000000000

Psocoptera 10111011101001110201011100011 1101?
01121101101100100000010110000001211101100000021300001000102200002010100010000 0001

Phthiraptera 111110111010011102010111000111101?0-11110------------00-0000000001211102-
000000213000000-0102200002010100000(01)0002000

Hemiptera 10111011101001014-
111111000011010011211011011001000000100000020012 1110(12)1000000215000000001022000
020101(01)0(01)(01)(01)0(01)00000

Neuroptera
101110111010010102010001000000110000114110110100010000211000000(23)111211102-
11000001310000000102200002 0111(01)0(01)000000000

Megaloptera
10111011101101010201000 10(01)0001100001141101101000 10000(01)111000002111211102-
11000001(16)1(01)000010102200002011110000100 0000

Raphidioptera 1011101110110101020100010000 01100?
0114110110100010000111100000(23)111211102-
1100000161(01)0000101022000020111000001000000

Coleoptera
101110111011010102010001000000110000114110110110013000111011000000 01211102-
01000001(37)1(01)0000101022000020111(01)00001000000

Strepsiptera 1(01)111011--1001010-
0100010000011000011411011011001010011101100000001211302---000000-1-??00(01)010?2?
00020111000001000000

Hymenoptera
101110111010010102010001 0(01)1001100001141101101100100000010(01)10002001211(01)0110
000000131000101110220000201110010000000(01)00

Trichoptera 10111011101001010-01----
00100110000114110111110010000001010000(23)001211102-
000000013111110111122000020111 10(01)(01)00000000

Lepidoptera

1011101110100101020100010010011000011411011111001000000100(01)000(123)001211102-000000013111110111122000020111001200000000

Mecoptera

1011101110100101020100010010011000011411011111001000100100(01)1012001210102-00000101(13)11111011102200012011100(01)000000000

Diptera *10111011101001010-11001100100110000114110111110010201001001000(12)001211102-000001013111111111022000020111(01)0(01)(02)000000(01)0*

Siphonaptera *11111011101001010-11101100100110000-14112--------------0111010001211102-00000101(13)100111-11022000120111000100000000*

;

END;

References

Beutel, R. G., & Gorb, S. N. (2001). Ultrastructure of attachment specializations of hexapods (Arthropoda): evolutionary patterns inferred from a revised ordinal phylogeny. Journal of Zoological Systematics and Evolutionary Research, 39, 177–207. https://doi.org/10.1046/j.1439-0469.2001.00155.x

Beutel, R. G., & Gorb, S. N. (2006). A revised interpretation of the evolution of attachment structures in Hexapoda with special emphasis on Mantophasmatodea. Arthropod Systematics & Phylogeny, 64, 3–25.

Blanke, A., Koch, M., Wipfler, B., Wilde, F., & Misof, B. (2014). Head morphology of *Tricholepidion gertschi* indicates monophyletic Zygentoma. Frontiers in Zoology, 11, 1–19. https://doi.org/10.1186/1742-9994-11-16

Cryan, J. R., & Urban, J. M. (2012). Higher-level phylogeny of the insect order Hemiptera: is Auchenorrhyncha really paraphyletic? Systematic Entomology, 37, 7–21. https://doi.org/10.1111/j.1365-3113.2011.00611.x

Flook, P. K., & Rowell, C. H. F. (1997). The effectiveness of mitochondrial rRNA sequences for the reconstruction of the phylogeny of an insect order (Orthoptera). Molecular Phylogenetics and Evolution, 8, 177–192. https://doi.org/10.1006/mpev.1997.0425

Flook, P. K., & Rowell, C. H. F. (1999). Combined molecular phylogenetic analysis of the Orthoptera (Arthropoda, Insecta) and implications for their higher systematics. Systematic Biology, 48, 233–253. https://doi.org/10.1080/106351599260274

Klass, K. D., Zompro, O., Kristensen, N. P., & Adis, J. (2002). Mantophasmatodea: a new insect order with extant members in the Afrotropics. Science, 296, 1456–1459. https://doi.org/10.1126/science.1069397

Song, H. (2010). Grasshopper systematics: past, present and future. Journal of Orthoptera Research, 19, 57–68. https://doi.org/10.1665/034.019.0112

Song, H. (2015). 300 million years of diversification: elucidating the patterns of orthopteran evolution based on comprehensive taxon and gene sampling. Cladistics, 31, 621–651. https://doi.org/10.1111/cla.12116

Song, F., Li, H., Jiang, P., Zhou, X., Liu, J., Sun, C., Vogler, A. P., & Cai, W. (2016). Capturing the phylogeny of Holometabola with mitochondrial genome data and Bayesian site-heterogeneous mixture model. Genome Biology and Evolution, 8, 1411–1426. https://doi.org/10.1093/gbe/evw086

Song, N., Liang, A.-P., & Bu, C.-P. (2012). A molecular phylogeny of Hemiptera inferred from mitochondrial genome sequences. PLoS ONE, 7, 1–13. https://doi.org/10.1371/journal.pone.0048778

Song, N., Zhang, H., & Zhao, T. (2019). Insights into the phylogeny of Hemiptera from increased mitogenomic taxon sampling. Molecular Phylogenetics and Evolution, 137, 236–249. https://doi.org/10.1016/j.ympev.2019.05.009

Terry, M. D., & Whiting, M. F. (2005). Mantophasmatodea and phylogeny of the lower neopterous insects. Cladistics, 21, 240–257. https://doi.org/10.1111/j.1096-0031.2005.00062.x

Wipfler, B., Machida, R., Müller, B., & Beutel, R. G. (2011). On the head morphology of Grylloblattodea (Insecta) and the systematic position of the order, with a new nomenclature for the head muscles of Dicondylia. Systematic Entomology, 36, 241–266. https://doi.org/10.1111/j.1365-3113.2010.00556.x

Zhang, H.-L., Huang, Y., Lin, L.-L., Wang, X.-Y., & Zheng, Z.-M. (2013). The phylogeny of the Orthoptera (Insecta) as deduced from mitogenomic gene sequences. Zoological Studies, 52, 1–13. https://doi.org/10.1186/1810-522X-52-37
