# Supplementary material for: Performance of tree-building methods using a morphological dataset and a well-supported Hexapoda phylogeny
Source: PeerJ. 2024 Jan 8;12:e16706. doi: 10.7717/peerj.16706 (PMC10782957; doi:10.7717/peerj.16706)
Supplement: Supplemental Information 2 [file peerj-12-16706-s002.pdf]

EW-MP (PAUP* 4.0a169 software):

```
#NEXUS
begin paup;
execute Beutel_Gorb_modified.txt;
log start file=Beutel_Gorb_modified_MP.log replace=yes;
begin assumptions;
options deftype=unord;
endblock;
set autoclose=yes criterion=parsimony increase=auto;
Pset msTaxa=variable;
hsearch start=stepwise nreps=10000 swap=tbr;
savetrees file=Beutel_Gorb_modified_MP.tre root=yes format=altnexus
maxdecimals=6 brlens=yes replace=yes;
contree all/strict=yes treefile=Beutel_Gorb_modified_MP_Consensus.tre;
pscores 1/ ci=yes RI=yes HI=yes RC=yes
scorefile=Beutel_Gorb_modified_MP_Scores.txt replace=yes;
Describetrees all/chglist Opt=acctran;
describe 1/diag;
bootstrap nreps=1000 conlevel=50 search=heuristic / nreps=10;
savetrees file=Beutel_Gorb_modified_MP_Bootstrap.tre root=yes format=altnexus
savebootp=both maxdecimals=6 from=1 to=1000 brlens=yes replace=yes;
log stop;
end;
```

IW-MP K=2 (PAUP* 4.0a169 software):

```
#NEXUS
begin paup;
execute Beutel_Gorb_modified.txt;
log start file=Beutel_Gorb_modified_MP.log replace=yes;
begin assumptions;
options deftype=unord;
endblock;
set autoclose=yes criterion=parsimony increase=auto;
Pset msTaxa=variable;
pset Goloboff=yes GK=2;
```

hsearch start=stepwise nreps=10000 swap=tbr;
savetrees file=Beutel_Gorb_modified_MP.tre root=yes format=altnexus
maxdecimals=6 brlens=yes replace=yes;
contree all/strict=yes treefile=Beutel_Gorb_modified_MP_Consensus.tre;
pscores 1/ ci=yes RI=yes HI=yes RC=yes
scorefile=Beutel_Gorb_modified_MP_Scores.txt replace=yes;
Describetrees all/chglist Opt=acctran;
describe 1/diag;
bootstrap nreps=1000 conlevel=50 search=heuristic / nreps=10;
savetrees file=Beutel_Gorb_modified_MP_Bootstrap.tre root=yes format=altnexus
savebootp=both maxdecimals=6 from=1 to=1000 brlens=yes replace=yes;
log stop;
end;

IW-MP K=3 (PAUP* 4.0a169 software):

#NEXUS
begin paup;
execute Beutel_Gorb_modified.txt;
log start file=Beutel_Gorb_modified_MP.log replace=yes;
begin assumptions;
options deftype=unord;
endblock;
set autoclose=yes criterion=parsimony increase=auto;
Pset msTaxa=variable;
pset Goloboff=yes GK=3;
hsearch start=stepwise nreps=10000 swap=tbr;
savetrees file=Beutel_Gorb_modified_MP.tre root=yes format=altnexus
maxdecimals=6 brlens=yes replace=yes;
contree all/strict=yes treefile=Beutel_Gorb_modified_MP_Consensus.tre;
pscores 1/ ci=yes RI=yes HI=yes RC=yes
scorefile=Beutel_Gorb_modified_MP_Scores.txt replace=yes;
Describetrees all/chglist Opt=acctran;
describe 1/diag;
bootstrap nreps=1000 conlevel=50 search=heuristic / nreps=10;
savetrees file=Beutel_Gorb_modified_MP_Bootstrap.tre root=yes format=altnexus

savebootp=both maxdecimals=6 from=1 to=1000 brlens=yes replace=yes;
log stop;
end;

IW-MP K=5 (PAUP* 4.0a169 software):

```
#NEXUS
begin paup;
execute Beutel_Gorb_modified.txt;
log start file=Beutel_Gorb_modified_MP.log replace=yes;
begin assumptions;
options deftype=unord;
endblock;
set autoclose=yes criterion=parsimony increase=auto;
Pset msTaxa=variable;
pset Goloboff=yes GK=5;
hsearch start=stepwise nreps=10000 swap=tbr;
savetrees file=Beutel_Gorb_modified_MP.tre root=yes format=altnexus
maxdecimals=6 brlens=yes replace=yes;
contree all/strict=yes treefile=Beutel_Gorb_modified_MP_Consensus.tre;
pscores 1/ ci=yes RI=yes HI=yes RC=yes
scorefile=Beutel_Gorb_modified_MP_Scores.txt replace=yes;
Describetrees all/chglist Opt=acctran;
describe 1/diag;
bootstrap nreps=1000 conlevel=50 search=heuristic / nreps=10;
savetrees file=Beutel_Gorb_modified_MP_Bootstrap.tre root=yes format=altnexus
savebootp=both maxdecimals=6 from=1 to=1000 brlens=yes replace=yes;
log stop;
end;
```

IW-MP K=10 (PAUP* 4.0a169 software):

```
#NEXUS
begin paup;
execute Beutel_Gorb_modified.txt;
log start file=Beutel_Gorb_modified_MP.log replace=yes;
begin assumptions;
```

```
options deftype=unord;
endblock;
set autoclose=yes criterion=parsimony increase=auto;
Pset msTaxa=variable;
pset Goloboff=yes GK=10;
hsearch start=stepwise nreps=10000 swap=tbr;
savetrees file=Beutel_Gorb_modified_MP.tre root=yes format=altnexus
maxdecimals=6 brlens=yes replace=yes;
contree all/strict=yes treefile=Beutel_Gorb_modified_MP_Consensus.tre;
pscores 1/ ci=yes RI=yes HI=yes RC=yes
scorefile=Beutel_Gorb_modified_MP_Scores.txt replace=yes;
Describetrees all/chglist Opt=acctran;
describe 1/diag;
bootstrap nreps=1000 conlevel=50 search=heuristic / nreps=10;
savetrees file=Beutel_Gorb_modified_MP_Bootstrap.tre root=yes format=altnexus
savebootp=both maxdecimals=6 from=1 to=1000 brlens=yes replace=yes;
log stop;
end;
```

IW-MP K=20 (PAUP* 4.0a169 software):

```
#NEXUS
begin paup;
execute Beutel_Gorb_modified.txt;
log start file=Beutel_Gorb_modified_MP.log replace=yes;
begin assumptions;
options deftype=unord;
endblock;
set autoclose=yes criterion=parsimony increase=auto;
Pset msTaxa=variable;
pset Goloboff=yes GK=20;
hsearch start=stepwise nreps=10000 swap=tbr;
savetrees file=Beutel_Gorb_modified_MP.tre root=yes format=altnexus
maxdecimals=6 brlens=yes replace=yes;
contree all/strict=yes treefile=Beutel_Gorb_modified_MP_Consensus.tre;
pscores 1/ ci=yes RI=yes HI=yes RC=yes
```

scorefile=Beutel_Gorb_modified_MP_Scores.txt replace=yes;
Describetrees all/chglist Opt=acctran;
describe 1/diag;
bootstrap nreps=1000 conlevel=50 search=heuristic / nreps=10;
savetrees file=Beutel_Gorb_modified_MP_Bootstrap.tre root=yes format=altnexus
savebootp=both maxdecimals=6 from=1 to=1000 brlens=yes replace=yes;
log stop;
end;

ML Mk (IQ-Tree 2 software):
iqtree2 -s Beutel_Gorb_modified.txt -nt AUTO -m MK -bb 10000 -minsup 0.5 -wbt -alrt 1000 -abayes -wsl

ML Mk+G (IQ-Tree 2 software):
iqtree2 -s Beutel_Gorb_modified.txt -nt AUTO -m MK+G -bb 10000 -minsup 0.5 -wbt -alrt 1000 -abayes -wsl

BI Mk (MrBayes 3.2.7a software):
begin mrbayes;
log start filename=Beutel_Gorb_modified_BI.log replace;
set autoclose=yes;
execute Beutel_Gorb_modified.txt;
lset rates=equal coding=all;
ctype Unordered:all;
mcmcp ngen=50000000 printfreq=10000 samplefreq=100
mcmcdiagn=yes diagnfreq=500000 stoprule=yes stopval=0.01
nchains=4 savebrlens=yes filename=Beutel_Gorb_modified_BI;
mcmc;
sumt contype=Halfcompat conformat=simple;
sump;
log stop;
end;

BI Mk+G (MrBayes 3.2.7a software):
begin mrbayes;

```
log start filename=Beutel_Gorb_modified_BI.log replace;
set autoclose=yes;
execute Beutel_Gorb_modified.txt;
lset rates=gamma coding=all;
ctype Unordered:all;
mcmcp ngen=50000000 printfreq=10000 samplefreq=100
mcmcdiagn=yes diagnfreq=500000 stoprule=yes stopval=0.01
nchains=4 savebrlens=yes filename=Beutel_Gorb_modified_BI;
mcmc;
sumt contype=Halfcompat conformat=simple;
sump;
log stop;
end;
```

Tree topology tests (PAUP* 4.0a169 software):

```
#NEXUS
begin paup;
execute Beutel_Gorb_modified.txt;
log start file=Beutel_Gorb_modified_Topology_tests.log replace=yes;
gettrees file=Trees.txt mode=7;
Pscores all /NonParamtest=yes ci=yes ri=yes;
Lscores all /KHtest=bootstrap RELL;
Lscores all /SHtest;
Lscores all /AUtest;
log stop;
end;
```
