# Supplementary material for: Performance of tree-building methods using a morphological dataset and a well-supported Hexapoda phylogeny
Source: PeerJ. 2024 Jan 8;12:e16706. doi: 10.7717/peerj.16706 (PMC10782957; doi:10.7717/peerj.16706)
Supplement: Supplemental Information 3 [file peerj-12-16706-s003.pdf]

Misof *et al.* (2014) reference tree
((Diplura,(Archaeognatha,(Zygentoma,((Ephemeroptera,Odonata),(((Plecoptera,(Orthoptera, ((Xenonomia,(Embioptera,Phasmatodea)),(Mantodea,(Blattodea,Isoptera))))), (Dermaptera,Zoraptera)),(((Psocoptera,Phthiraptera),(Hymenoptera,(((Raphidioptera, (Megaloptera,Neuroptera)),(Coleoptera,Strepsiptera)),((Diptera,(Siphonaptera,Mecoptera)), (Trichoptera,Lepidoptera))))),(Thysanoptera,Hemiptera))))))),(Collembola,Protura));
