# Supplementary material for: Performance of tree-building methods using a morphological dataset and a well-supported Hexapoda phylogeny
Source: PeerJ. 2024 Jan 8;12:e16706. doi: 10.7717/peerj.16706 (PMC10782957; doi:10.7717/peerj.16706)
Supplement: Supplemental Information 4 [file peerj-12-16706-s004.pdf]

Equal-weight maximum parsimony (EW-MP); 50% threshold tree; nonparametric bootstrap
(Diplura:100,(Protura:100,Collembola:100)99.334:99.334,(Archaeognatha:100,(Zygentoma:100,(Ephemeroptera:100,(Odonata:100,(Plecoptera:100,(Dermaptera:100,Embioptera:100)61.202:61.202,((Blattodea:100,Isoptera:100)52.203:52.203,Mantodea:100)64.97:64.97,Orthoptera:100,Phasmatodea:100,Xenonomia:100,(Zoraptera:100,((Thysanoptera:100,(Psocoptera:100,Phthiraptera:100)68.667:68.667,Hemiptera:100)73.347:73.347,(((Neuroptera:100,(Megaloptera:100,Raphidioptera:100)95.5:95.5)90.111:90.111,(Coleoptera:100,Strepsiptera:100)70.183:70.183)76.107:76.107,(Hymenoptera:100,((Trichoptera:100,Lepidoptera:100)66.592:66.592,(Mecoptera:100,Diptera:100,Siphonaptera:100)62.19:62.19)63.065:63.065)91.666:91.666)90.254:90.254)77.953:77.953)60.708:60.708)91.989:91.989)86.817:86.817)86.859:86.859)99.699:99.699)100:100);

Implied-weight maximum parsimony (IW-MP K=2); 50% threshold tree; nonparametric bootstrap
(Diplura:100,(Protura:100,Collembola:100)99.867:99.867,(Archaeognatha:100,(Zygentoma:100,(Ephemeroptera:100,(Odonata:100,(Plecoptera:100,(Dermaptera:100,Embioptera:100)57.584:57.584,((Blattodea:100,Isoptera:100)56.099:56.099,Mantodea:100)77.637:77.637,Orthoptera:100,Phasmatodea:100,Xenonomia:100,Zoraptera:100,((Thysanoptera:100,(Psocoptera:100,Phthiraptera:100)88.264:88.264,Hemiptera:100)82.469:82.469,(((Neuroptera:100,(Megaloptera:100,Raphidioptera:100)95.386:95.386)88.234:88.234,(Coleoptera:100,Strepsiptera:100)70.678:70.678)91.514:91.514,(Hymenoptera:100,((Trichoptera:100,Lepidoptera:100)72.48:72.48,((Mecoptera:100,Siphonaptera:100)67.137:67.137,Diptera:100)88.412:88.412)96.036:96.036)96.737:96.737)93.804:93.804)84.993:84.993)97.017:97.017)95.974:95.974)82.356:82.356)99.833:99.833)100:100);

Implied-weight maximum parsimony (IW-MP K=3); 50% threshold tree; nonparametric bootstrap
(Diplura:100,(Protura:100,Collembola:100)99:99,(Archaeognatha:100,(Zygentoma:100,(Ephemeroptera:100,(Odonata:100,(Plecoptera:100,(Dermaptera:100,Embioptera:100)57.661:57.661,((Blattodea:100,Isoptera:100)53.59:53.59,Mantodea:100)74.7:74.7,Orthoptera:100,Phasmatodea:100,Xenonomia:100,Zoraptera:100,((Thysanoptera:100,(Psocoptera:100,Phthiraptera:100)89.207:89.207,Hemiptera:100)83.92:83.92,(((Neuroptera:100,(Megaloptera:100,Raphidioptera:100)94.565:94.565)87.213:87.213,(Coleoptera:100,Strepsiptera:100)68.814:68.814)90.543:90.543,(Hymenoptera:100,((Trichoptera:100,Lepidoptera:100)73.439:73.439,((Mecoptera:100,Siphonaptera:100)64.2:64.2,Diptera:100)86.091:86.091)95.32:95.32)97.132:97.132)93.538:93.538)84.955:84.955)96.909:96.909)93.99:93.99)81.677:81.677)99.8:99.8)100:100);

Implied-weight maximum parsimony (IW-MP K=5); 50% threshold tree; nonparametric bootstrap
(Diplura:100,(Protura:100,Collembola:100)99.667:99.667,(Archaeognatha:100,(Zygentoma:100,(Ephemeroptera:100,(Odonata:100,(Plecoptera:100,(Dermaptera:100,Embioptera:100)62.96:62.96,((Blattodea:100,Isoptera:100)52.984:52.984,Mantodea:100)71.867:71.867,Orthoptera:100,Phasmatodea:100,Xenonomia:100,(Zoraptera:100,((Thysanoptera:100,(Psocoptera:100,Phthiraptera:100)88.096:88.096,Hemiptera:100)83.165:83.165,(((Neuroptera:100,(Megaloptera:100,Raphidioptera:100)95.057:95.057)87.461:87.461,(Coleoptera:100,Strepsiptera:100)70.981:70.981)90.801:90.801,(Hymenoptera:100,

((Trichoptera:100,Lepidoptera:100)73.567:73.567,
((Mecoptera:100,Siphonaptera:100)61.451:61.451,Diptera:100)85.096:85.096)91.973:91.973)96.39:96.39)94.754:94.754)85.486:85.486)50.259:50.259)95.891:95.891)94.249:94.249)81.638:81.638)99.9:99.9)100:100);

Implied-weight maximum parsimony (IW-MP K=10); 50% threshold tree; nonparametric bootstrap
(Diplura:100,(Protura:100,Collembola:100)99.333:99.333,(Archaeognatha:100,(Zygentoma:100,
(Ephemeroptera:100,(Odonata:100,(Plecoptera:100,
(Dermaptera:100,Embioptera:100)61.889:61.889,
((Blattodea:100,Isoptera:100)51.366:51.366,Mantodea:100)70.1:70.1,Orthoptera:100,Phasmatodea:100,Xenonomia:100,Zoraptera:100,((Thysanoptera:100,
(Psocoptera:100,Phthiraptera:100)83.377:83.377,Hemiptera:100)84.282:84.282,(((Neuroptera:100,
(Megaloptera:100,Raphidioptera:100)95.818:95.818)86.972:86.972,
(Coleoptera:100,Strepsiptera:100)70.723:70.723)88.468:88.468,(Hymenoptera:100,
((Trichoptera:100,Lepidoptera:100)72.688:72.688,
((Mecoptera:100,Siphonaptera:100)51.948:51.948,Diptera:100)81.267:81.267)90.799:90.799)95.333:95.333)94.112:94.112)82.565:82.565)96.846:96.846)95.065:95.065)85.603:85.603)99.7:99.7)100:100);

Implied-weight maximum parsimony (IW-MP K=20); 50% threshold tree; nonparametric bootstrap
(Diplura:100,(Protura:100,Collembola:100)99.4:99.4,(Archaeognatha:100,(Zygentoma:100,
(Ephemeroptera:100,(Odonata:100,(Plecoptera:100,
(Dermaptera:100,Embioptera:100)61.301:61.301,
(Blattodea:100,Isoptera:100,Mantodea:100)69.487:69.487,Orthoptera:100,Phasmatodea:100,Xenonomia:100,(Zoraptera:100,((Thysanoptera:100,
(Psocoptera:100,Phthiraptera:100)81.283:81.283,Hemiptera:100)80.394:80.394,(((Neuroptera:100,
(Megaloptera:100,Raphidioptera:100)95.809:95.809)86.319:86.319,
(Coleoptera:100,Strepsiptera:100)69.148:69.148)82.924:82.924,(Hymenoptera:100,
((Trichoptera:100,Lepidoptera:100)71.616:71.616,
(Mecoptera:100,Diptera:100,Siphonaptera:100)78.938:78.938)83.965:83.965)95.701:95.701)92.968:92.968)81.199:81.199)55.93:55.93)94.772:94.772)92.139:92.139)82.163:82.163)99.9:99.9)100:100);

Maximum likelihood, Mk model (ML Mk); 50% threshold tree; "ultrafast" bootstrap
(Diplura:0.0086357112,(Protura:0.0496776249,Collembola:0.0519545673)100:0.1071226760,
(Archaeognatha:0.0086650719,(Zygentoma:0.0333109368,(Ephemeroptera:0.0477940857,
(Odonata:0.0216416984,(Plecoptera:0.0283360045,
((Dermaptera:0.0588124151,Embioptera:0.0332032389)74:0.0301377042,
(Zoraptera:0.0158732015,
(((Thysanoptera:0.0520196380,Hemiptera:0.0687346328)59:0.0026402269,
(Psocoptera:0.0592082419,Phthiraptera:0.0266333805)93:0.0272630606)92:0.0359987141,
(((Neuroptera:0.0236233023,
(Megaloptera:0.0100413773,Raphidioptera:0.0000020668)99:0.0340898283)90:0.0481621105,
(Coleoptera:0.0183247008,Strepsiptera:0.0304468395)88:0.0158363685)85:0.0324120836,
(Hymenoptera:0.0099058623,
((Trichoptera:0.0238615265,Lepidoptera:0.0000020723)98:0.0222989108,
(Mecoptera:0.0375669144,
(Diptera:0.0000020836,Siphonaptera:0.1261946378)68:0.0440229766)87:0.0188542413)90:0.0364638756)99:0.0449744094)98:0.0414339736)96:0.0295109897)85:0.0329324009)55:0.0267498030,
((((Blattodea:0.0000020668,Isoptera:0.0450216112)78:0.0175697564,Mantodea:0.0000020668)77:

0.0414329288,Phasmatodea:0.0300653166)52:0.0086679110,Orthoptera:0.0389394728,Xenonomia:0.0104899003)76:0.0466883146)97:0.0547824649)95:0.0579200092)97:0.0600606109)100:0.1380605462)100:0.1822813843);

Maximum likelihood, Mk+Gamma model (ML Mk+G); 50% threshold tree; "ultrafast" bootstrap

(Diplura:0.0175701613,(Protura:0.0446197388,Collembola:0.0538293439)100:0.1112670956,
(Archaeognatha:0.0264921750,(Zygentoma:0.0355080357,(Ephemeroptera:0.0631389777,
(Odonata:0.0355080357,((Plecoptera:0.0446197388,
((((Blattodea:0.0087400612,Isoptera:0.0446197388)100:0.0175701613,Mantodea:0.0087400612)100:0.0446197388,Phasmatodea:0.0355080357)61:0.0175701613,Xenonomia:0.0087400612,Orthoptera:0.0446197388)80:0.0538293439)56:0.0355080357,
(Dermaptera:0.0631389777,Embioptera:0.0446197388)91:0.0446197388,(Zoraptera:0.0264921750,
((Thysanoptera:0.0538293439,
((Psocoptera:0.0631389777,Phthiraptera:0.0355080357)100:0.0264921750,Hemiptera:0.0725508370)62:0.0264921750)100:0.0355080357,(((Neuroptera:0.0264921750,
(Megaloptera:0.0175701613,Raphidioptera:0.0087400612)100:0.0446197388)100:0.0538293439,
(Coleoptera:0.0175701613,Strepsiptera:0.0264921750)80:0.0264921750)100:0.0355080357,
(Hymenoptera:0.0264921750,
((Trichoptera:0.0264921750,Lepidoptera:0.0175701613)100:0.0355080357,
((Mecoptera:0.0446197388,Siphonaptera:0.1112670956)55:0.0355080357,Diptera:0.0355080357)100:0.0538293439)100:0.0355080357)100:0.0446197388)100:0.0446197388)98:0.0355080357)92:0.0538293439)100:0.0725508370)100:0.0725508370)100:0.0725508370)100:0.1212257138)100:0.1518151962);

Bayesian inference, Mk model (BI Mk); 50% threshold tree; posterior probability

(Diplura:1.167497e-002,(Archaeognatha:1.266231e-002,(Zygentoma:3.012744e-002,
(Ephemeroptera:4.700683e-002,(Odonata:2.203288e-002,(Plecoptera:3.054235e-002,
((Dermaptera:5.195517e-002,Embioptera:3.124940e-002)0.893:2.953477e-002,
(Zoraptera:1.910280e-002,(((Thysanoptera:4.311822e-002,Hemiptera:5.431950e-002)0.501:1.317638e-002,(Psocoptera:4.958457e-002,Phthiraptera:2.849347e-002)0.945:2.507798e-002)0.993:3.292666e-002,(((Neuroptera:2.218114e-002,
(Megaloptera:1.102576e-002,Raphidioptera:5.002016e-003)0.998:2.923989e-002)0.999:4.728090e-002,(Coleoptera:1.886900e-002,Strepsiptera:2.847155e-002)0.805:1.990708e-002)0.939:3.108681e-002,(Hymenoptera:1.263123e-002,
((Trichoptera:2.310351e-002,Lepidoptera:1.222528e-002)0.827:1.435371e-002,
(Mecoptera:2.880622e-002,(Diptera:1.051533e-002,Siphonaptera:9.702455e-002)0.847:3.422660e-002)0.935:2.232283e-002)0.985:3.442699e-002)0.998:4.002595e-002)0.998:3.799524e-002)0.985:2.782235e-002)0.972:3.009846e-002)0.752:2.419424e-002,((((Blattodea:4.959963e-003,Isoptera:3.935200e-002)0.932:1.606938e-002,Mantodea:6.150043e-003)0.975:3.774067e-002,Phasmatodea:2.868002e-002)0.550:1.348522e-002,Orthoptera:3.397136e-002,Xenonomia:1.248048e-002)0.984:3.651041e-002)0.997:4.765777e-002)0.996:5.072965e-002)0.990:4.921760e-002)1.000:1.102664e-001)1.000:1.447727e-001,(Protura:4.548803e-002,Collembola:4.310525e-002)1.000:8.125336e-002);

Bayesian inference, Mk+Gamma model (BI Mk+G); 50% threshold tree; posterior probability

(Diplura:8.529287e-003,(Archaeognatha:1.079274e-002,(Zygentoma:2.503885e-002,
(Ephemeroptera:3.538683e-002,(Odonata:1.575855e-002,(Plecoptera:2.291804e-002,
((Dermaptera:3.924958e-002,Embioptera:2.490717e-002)0.912:2.314403e-002,
(Zoraptera:1.444769e-002,(((Thysanoptera:3.181707e-002,Hemiptera:4.323232e-002)0.523:1.054509e-002,(Psocoptera:3.675961e-002,Phthiraptera:2.050499e-

002)0.976:1.960351e-002)0.996:2.616267e-002,(((Neuroptera:1.736686e-002,
(Megaloptera:7.925403e-003,Raphidioptera:4.158572e-003)0.998:2.160837e-
002)0.999:3.648047e-002,(Coleoptera:1.402581e-002,Strepsiptera:2.174755e-
002)0.697:1.410069e-002)0.971:2.495976e-002,(Hymenoptera:8.983908e-003,
((Trichoptera:1.726929e-002,Lepidoptera:8.947041e-003)0.887:1.136626e-002,
(Mecoptera:1.724138e-002,(Diptera:1.289609e-002,Siphonaptera:7.579034e-002)0.577:2.601004e-
002)0.972:1.749295e-002)0.997:2.577516e-002)0.999:3.068173e-002)0.997:2.933225e-
002)0.957:2.057395e-002)0.966:2.444116e-002)0.714:1.814010e-002,((((Blattodea:3.797281e-
003,Isoptera:2.984914e-002)0.905:1.188054e-002,Mantodea:4.965124e-003)1.000:2.905629e-
002,Phasmatodea:2.065843e-002)0.616:1.031391e-002,Orthoptera:2.599257e-
002,Xenonomia:9.023293e-003)0.981:2.793943e-002)0.998:3.764004e-002)0.999:3.895770e-
002)0.990:3.718490e-002)1.000:8.447638e-002)1.000:1.119896e-001,(Protura:3.504991e-
002,Collembola:3.185117e-002)1.000:6.480184e-002);
