# Supplementary material for: Performance of tree-building methods using a morphological dataset and a well-supported Hexapoda phylogeny
Source: PeerJ. 2024 Jan 8;12:e16706. doi: 10.7717/peerj.16706 (PMC10782957; doi:10.7717/peerj.16706)
Supplement: Supplemental Information 7 [file peerj-12-16706-s007.pdf]

Equal-weight maximum parsimony (EW-MP); 95% threshold tree; nonparametric bootstrap
(Diplura:100,(Protura:100,Collembola:100)99.334:99.334,(Archaeognatha:100,(Zygentoma:100,Ephemeroptera:100,Odonata:100,Plecoptera:100,Dermaptera:100,Embioptera:100,Blattodea:100,Isoptera:100,Mantodea:100,Orthoptera:100,Phasmatodea:100,Xenonomia:100,Zoraptera:100,Thysanoptera:100,Psocoptera:100,Phthiraptera:100,Hemiptera:100,Neuroptera:100,(Megaloptera:100,Raphidioptera:100)95.5:95.5,Coleoptera:100,Strepsiptera:100,Hymenoptera:100,Trichoptera:100,Lepidoptera:100,Mecoptera:100,Diptera:100,Siphonaptera:100)99.699:99.699)100:100);

Implied-weight maximum parsimony (IW-MP K=2); 95% threshold tree; nonparametric bootstrap
(Diplura:100,(Protura:100,Collembola:100)99.867:99.867,(Archaeognatha:100,(Zygentoma:100,Ephemeroptera:100,(Odonata:100,(Plecoptera:100,Dermaptera:100,Embioptera:100,Blattodea:100,Isoptera:100,Mantodea:100,Orthoptera:100,Phasmatodea:100,Xenonomia:100,Zoraptera:100,Thysanoptera:100,Psocoptera:100,Phthiraptera:100,Hemiptera:100,Neuroptera:100,(Megaloptera:100,Raphidioptera:100)95.386:95.386,Coleoptera:100,Strepsiptera:100,(Hymenoptera:100,(Trichoptera:100,Lepidoptera:100,Mecoptera:100,Siphonaptera:100,Diptera:100)96.036:96.036)96.737:96.737)97.017:97.017)95.974:95.974)99.833:99.833)100:100);

Implied-weight maximum parsimony (IW-MP K=3); 95% threshold tree; nonparametric bootstrap
(Diplura:100,(Protura:100,Collembola:100)99:99,(Archaeognatha:100,(Zygentoma:100,Ephemeroptera:100,Odonata:100,(Plecoptera:100,Dermaptera:100,Embioptera:100,Blattodea:100,Isoptera:100,Mantodea:100,Orthoptera:100,Phasmatodea:100,Xenonomia:100,Zoraptera:100,Thysanoptera:100,Psocoptera:100,Phthiraptera:100,Hemiptera:100,Neuroptera:100,Megaloptera:100,Raphidioptera:100,Coleoptera:100,Strepsiptera:100,(Hymenoptera:100,(Trichoptera:100,Lepidoptera:100,Mecoptera:100,Siphonaptera:100,Diptera:100)95.32:95.32)97.132:97.132)96.909:96.909)99.8:99.8)100:100);

Implied-weight maximum parsimony (IW-MP K=5); 95% threshold tree; nonparametric bootstrap
(Diplura:100,(Protura:100,Collembola:100)99.667:99.667,(Archaeognatha:100,(Zygentoma:100,Ephemeroptera:100,Odonata:100,(Plecoptera:100,Dermaptera:100,Embioptera:100,Blattodea:100,Isoptera:100,Mantodea:100,Orthoptera:100,Phasmatodea:100,Xenonomia:100,Zoraptera:100,Thysanoptera:100,Psocoptera:100,Phthiraptera:100,Hemiptera:100,Neuroptera:100,(Megaloptera:100,Raphidioptera:100)95.057:95.057,Coleoptera:100,Strepsiptera:100,(Hymenoptera:100,Trichoptera:100,Lepidoptera:100,Mecoptera:100,Siphonaptera:100,Diptera:100)96.39:96.39)95.891:95.891)99.9:99.9)100:100);

Implied-weight maximum parsimony (IW-MP K=10); 95% threshold tree; nonparametric bootstrap
(Diplura:100,(Protura:100,Collembola:100)99.333:99.333,(Archaeognatha:100,(Zygentoma:100,Ephemeroptera:100,(Odonata:100,(Plecoptera:100,Dermaptera:100,Embioptera:100,Blattodea:100,Isoptera:100,Mantodea:100,Orthoptera:100,Phasmatodea:100,Xenonomia:100,Zoraptera:100,Thysanoptera:100,Psocoptera:100,Phthiraptera:100,Hemiptera:100,Neuroptera:100,(Megaloptera:100,Raphidioptera:100)95.818:95.818,Coleoptera:100,Strepsiptera:100,

(Hymenoptera:100,Trichoptera:100,Lepidoptera:100,Mecoptera:100,Siphonaptera:100,Diptera:100)
95.333:95.333)96.846:96.846)95.065:95.065)99.7:99.7)100:100);

Implied-weight maximum parsimony (IW-MP K=20); 95% threshold tree; nonparametric bootstrap

(Diplura:100,(Protura:100,Collembola:100)99.4:99.4,(Archaeognatha:100,
(Zygentoma:100,Ephemeroptera:100,Odonata:100,Plecoptera:100,Dermaptera:100,Embioptera:100,
Blattodea:100,Isoptera:100,Mantodea:100,Orthoptera:100,Phasmatodea:100,Xenonomia:100,Zorapt
era:100,Thysanoptera:100,Psocoptera:100,Phthiraptera:100,Hemiptera:100,Neuroptera:100,
(Megaloptera:100,Raphidioptera:100)95.809:95.809,Coleoptera:100,Strepsiptera:100,
(Hymenoptera:100,Trichoptera:100,Lepidoptera:100,Mecoptera:100,Diptera:100,Siphonaptera:100)
95.701:95.701)99.9:99.9)100:100);

Maximum likelihood, Mk model (ML Mk); 95% threshold tree; "ultrafast" bootstrap

(Diplura:0.0086357112,(Protura:0.0496776249,Collembola:0.0519545673)100:0.1071226760,
(Archaeognatha:0.0086650719,(Zygentoma:0.0333109368,(Ephemeroptera:0.0477940857,
(Odonata:0.0216416984,
(Plecoptera:0.0283360045,Dermaptera:0.0588124151,Embioptera:0.0332032389,Zoraptera:0.0158
732015,
(Thysanoptera:0.0520196380,Hemiptera:0.0687346328,Psocoptera:0.0592082419,Phthiraptera:0.02
66333805,(Neuroptera:0.0236233023,
(Megaloptera:0.0100413773,Raphidioptera:0.0000020668)99:0.0340898283,Coleoptera:0.0183247
008,Strepsiptera:0.0304468395,(Hymenoptera:0.0099058623,
(Trichoptera:0.0238615265,Lepidoptera:0.0000020723)98:0.0222989108,Mecoptera:0.0375669144
,Diptera:0.0000020836,Siphonaptera:0.1261946378)99:0.0449744094)98:0.0414339736)96:0.0295
109897,Blattodea:0.0000020668,Isoptera:0.0450216112,Mantodea:0.0000020668,Phasmatodea:0.0
300653166,Orthoptera:0.0389394728,Xenonomia:0.0104899003)97:0.0547824649)95:0.05792000
92)97:0.0600606109)100:0.1380605462)100:0.1822813843);

Maximum likelihood, Mk+Gamma model (ML Mk+G); 95% threshold tree; "ultrafast" bootstrap

(Diplura:0.0175701613,(Protura:0.0446197388,Collembola:0.0538293439)100:0.1112670956,
(Archaeognatha:0.0264921750,(Zygentoma:0.0355080357,(Ephemeroptera:0.0631389777,
(Odonata:0.0355080357,(Plecoptera:0.0446197388,
((Blattodea:0.0087400612,Isoptera:0.0446197388)100:0.0175701613,Mantodea:0.0087400612)100
:0.0446197388,Phasmatodea:0.0355080357,Xenonomia:0.0087400612,Orthoptera:0.0446197388,D
ermaptera:0.0631389777,Embioptera:0.0446197388,Zoraptera:0.0264921750,
((Thysanoptera:0.0538293439,
(Psocoptera:0.0631389777,Phthiraptera:0.0355080357)100:0.0264921750,Hemiptera:0.072550837
0)100:0.0355080357,(((Neuroptera:0.0264921750,
(Megaloptera:0.0175701613,Raphidioptera:0.0087400612)100:0.0446197388)100:0.0538293439,C
oleoptera:0.0175701613,Strepsiptera:0.0264921750)100:0.0355080357,
(Hymenoptera:0.0264921750,
((Trichoptera:0.0264921750,Lepidoptera:0.0175701613)100:0.0355080357,
(Mecoptera:0.0446197388,Siphonaptera:0.1112670956,Diptera:0.0355080357)100:0.0538293439)1
00:0.0355080357)100:0.0446197388)100:0.0446197388)98:0.0355080357)100:0.0725508370)100:
0.0725508370)100:0.0725508370)100:0.1212257138)100:0.1518151962);

Bayesian inference, Mk model (BI Mk); 95% threshold tree; posterior probability

(Diplura:1.167497e-002,(Archaeognatha:1.266231e-002,(Zygentoma:3.012744e-002,
(Ephemeroptera:4.700683e-002,(Odonata:2.203288e-002,(Plecoptera:3.054235e-
002,Dermaptera:5.195517e-002,Embioptera:3.124940e-002,(Zoraptera:1.910280e-002,

((Thysanoptera:4.311822e-002,Hemiptera:5.431950e-002,Psocoptera:4.958457e-002,Phthiraptera:2.849347e-002)0.993:3.292666e-002,((Neuroptera:2.218114e-002,(Megaloptera:1.102576e-002,Raphidioptera:5.002016e-003)0.998:2.923989e-002)0.999:4.728090e-002,Coleoptera:1.886900e-002,Strepsiptera:2.847155e-002,(Hymenoptera:1.263123e-002,(Trichoptera:2.310351e-002,Lepidoptera:1.222528e-002,Mecoptera:2.880622e-002,Diptera:1.051533e-002,Siphonaptera:9.702455e-002)0.985:3.442699e-002)0.998:4.002595e-002)0.998:3.799524e-002)0.985:2.782235e-002)0.972:3.009846e-002,((Blattodea:4.959963e-003,Isoptera:3.935200e-002,Mantodea:6.150043e-003)0.975:3.774067e-002,Phasmatodea:2.868002e-002,Orthoptera:3.397136e-002,Xenonomia:1.248048e-002)0.984:3.651041e-002)0.997:4.765777e-002)0.996:5.072965e-002)0.990:4.921760e-002)1.000:1.102664e-001)1.000:1.447727e-001,(Protura:4.548803e-002,Collembola:4.310525e-002)1.000:8.125336e-002);

Bayesian inference, Mk+Gamma model (BI Mk+G); 95% threshold tree; posterior probability

(Diplura:8.529287e-003,(Archaeognatha:1.079274e-002,(Zygentoma:2.503885e-002,(Ephemeroptera:3.538683e-002,(Odonata:1.575855e-002,(Plecoptera:2.291804e-002,Dermaptera:3.924958e-002,Embioptera:2.490717e-002,(Zoraptera:1.444769e-002,((Thysanoptera:3.181707e-002,Hemiptera:4.323232e-002,(Psocoptera:3.675961e-002,Phthiraptera:2.050499e-002)0.976:1.960351e-002)0.996:2.616267e-002,(((Neuroptera:1.736686e-002,(Megaloptera:7.925403e-003,Raphidioptera:4.158572e-003)0.998:2.160837e-002)0.999:3.648047e-002,Coleoptera:1.402581e-002,Strepsiptera:2.174755e-002)0.971:2.495976e-002,(Hymenoptera:8.983908e-003,(Trichoptera:1.726929e-002,Lepidoptera:8.947041e-003,(Mecoptera:1.724138e-002,Diptera:1.289609e-002,Siphonaptera:7.579034e-002)0.972:1.749295e-002)0.997:2.577516e-002)0.999:3.068173e-002)0.997:2.933225e-002)0.957:2.057395e-002)0.966:2.444116e-002,((Blattodea:3.797281e-003,Isoptera:2.984914e-002,Mantodea:4.965124e-003)1.000:2.905629e-002,Phasmatodea:2.065843e-002,Orthoptera:2.599257e-002,Xenonomia:9.023293e-003)0.981:2.793943e-002)0.998:3.764004e-002)0.999:3.895770e-002)0.990:3.718490e-002)1.000:8.447638e-002)1.000:1.119896e-001,(Protura:3.504991e-002,Collembola:3.185117e-002)1.000:6.480184e-002);
