# Supplementary figures and images for: Performance of tree-building methods using a morphological dataset and a well-supported Hexapoda phylogeny
Source: PeerJ. 2024 Jan 8;12:e16706. doi: 10.7717/peerj.16706 (PMC10782957; doi:10.7717/peerj.16706)

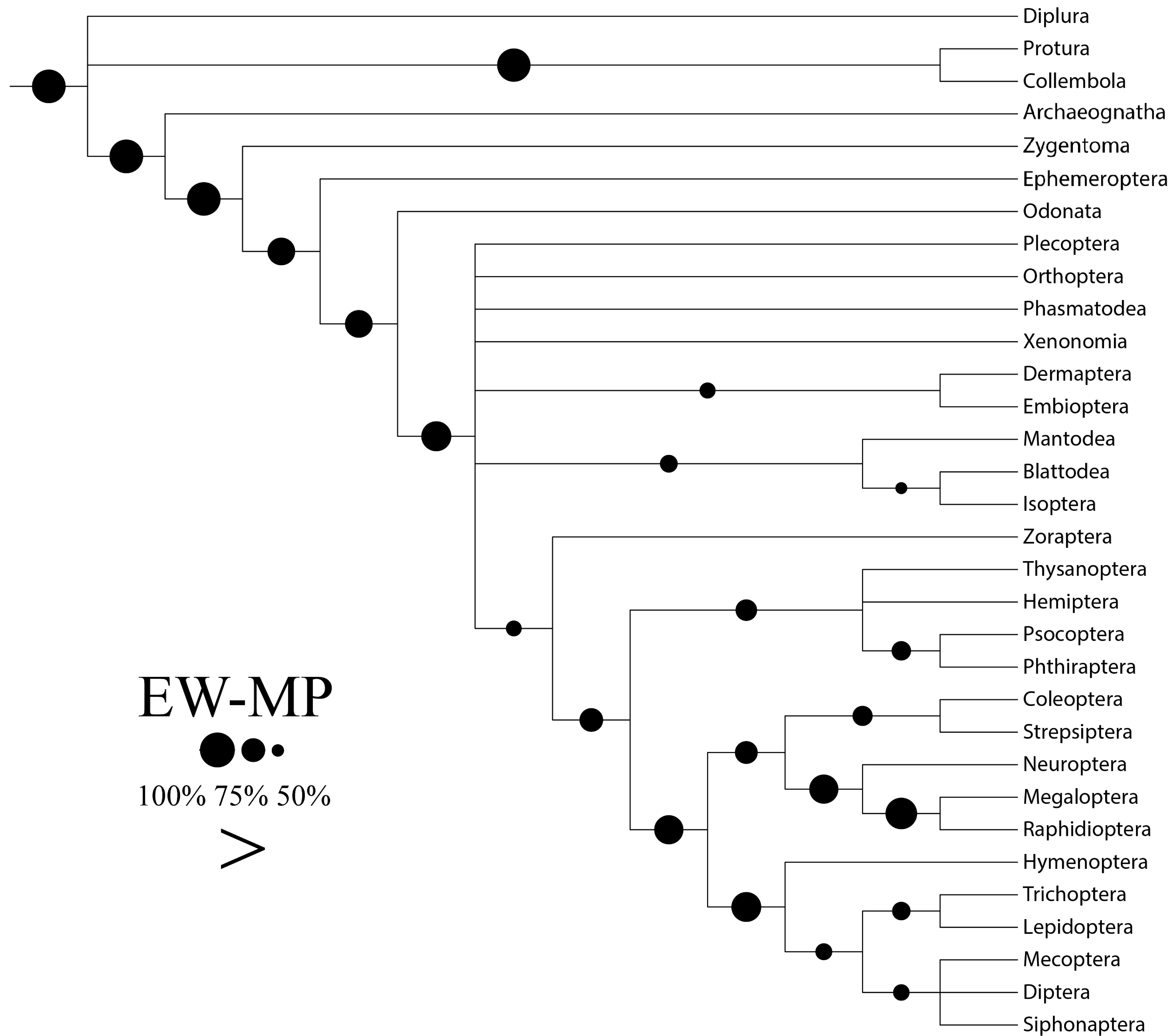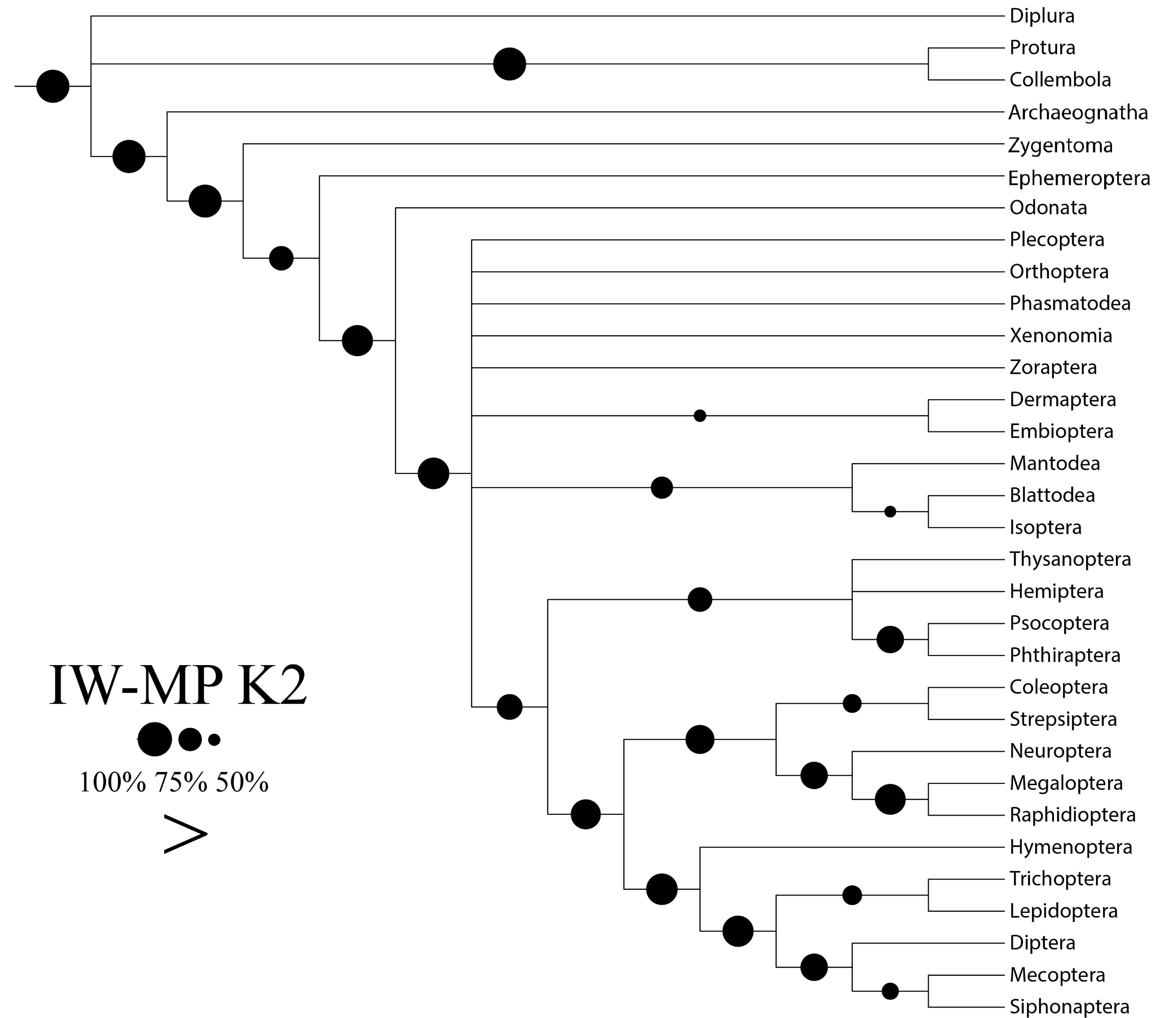

Supplement: Supplemental Information 8 [file peerj-12-16706-s008.pdf]

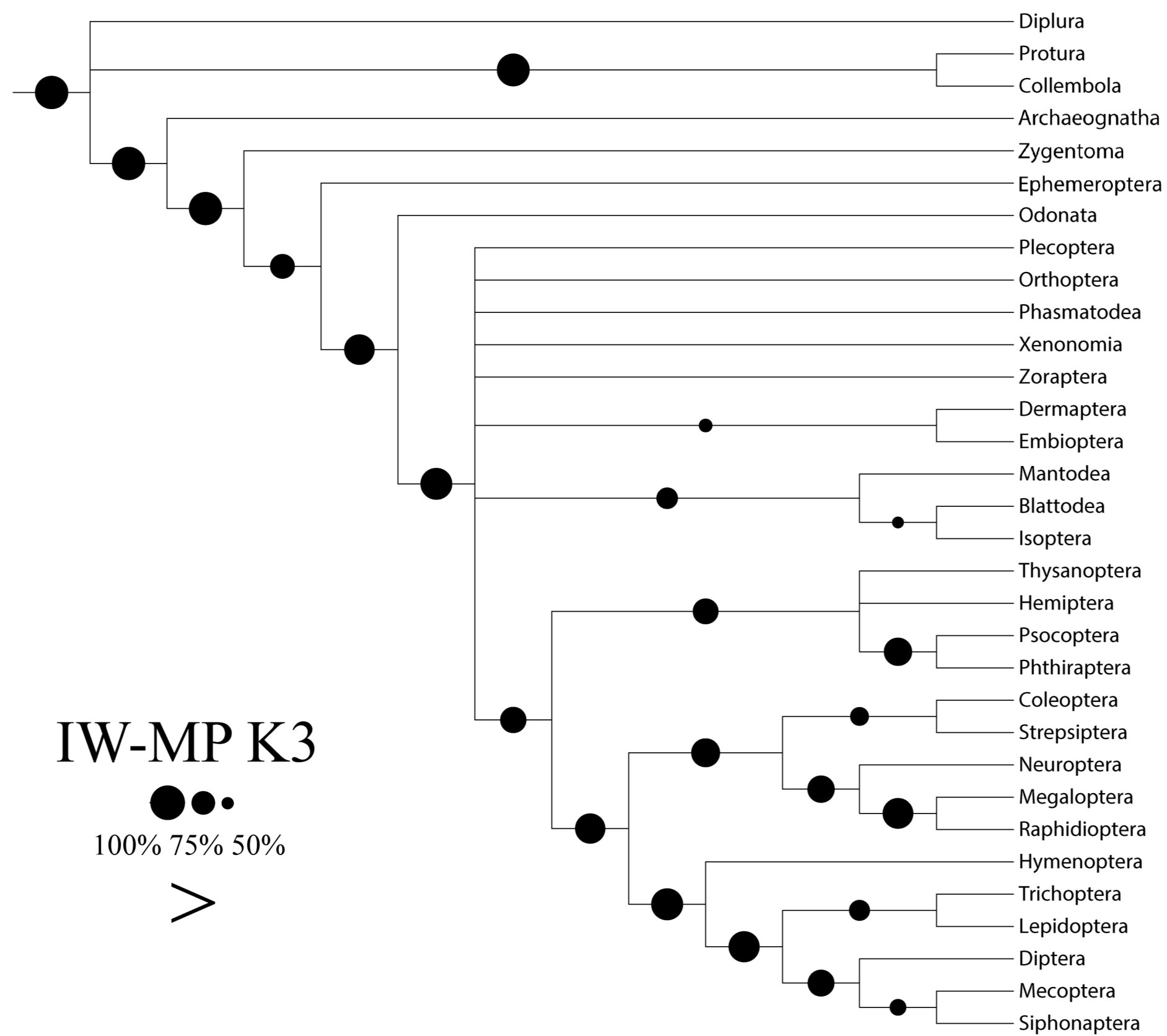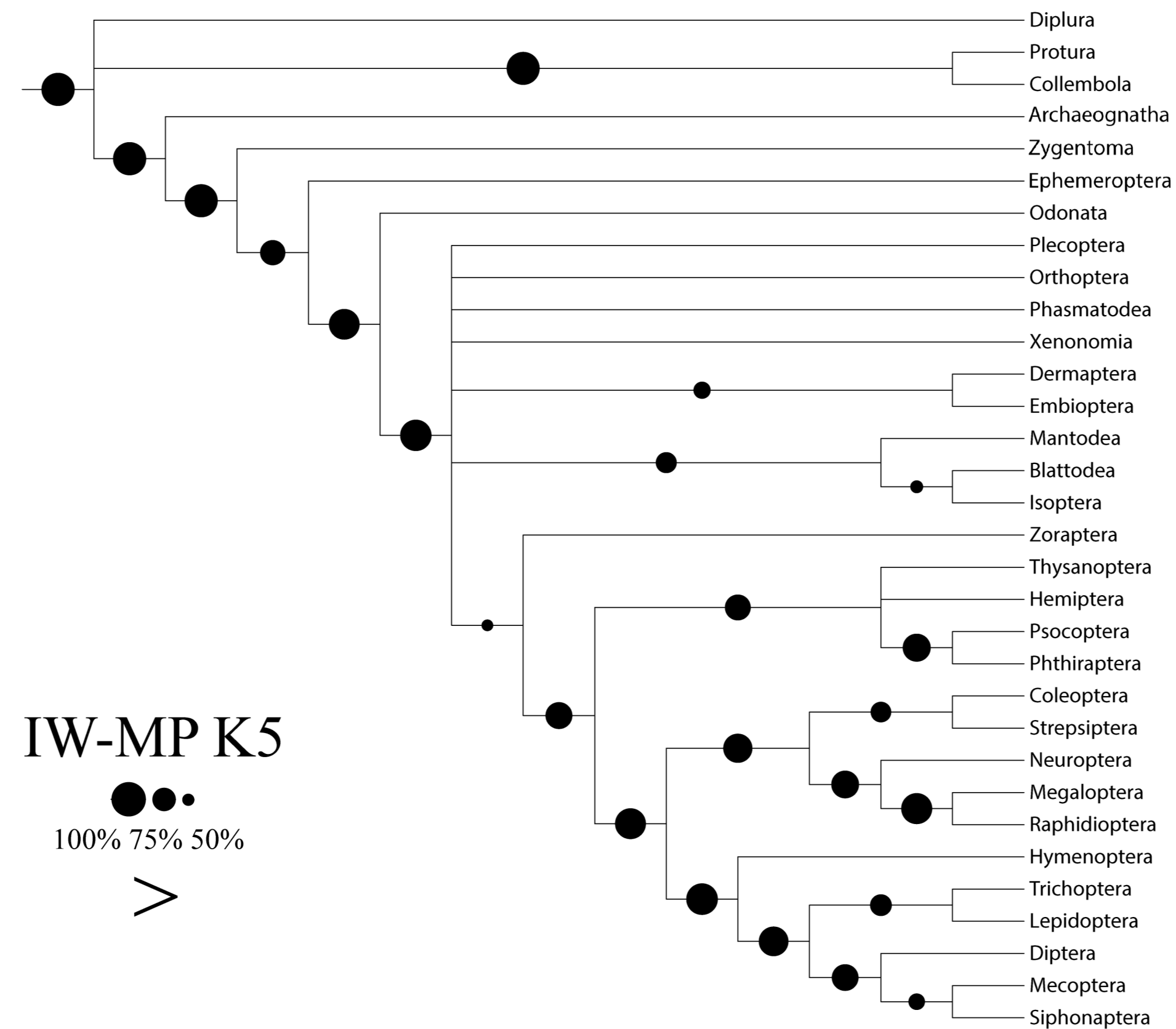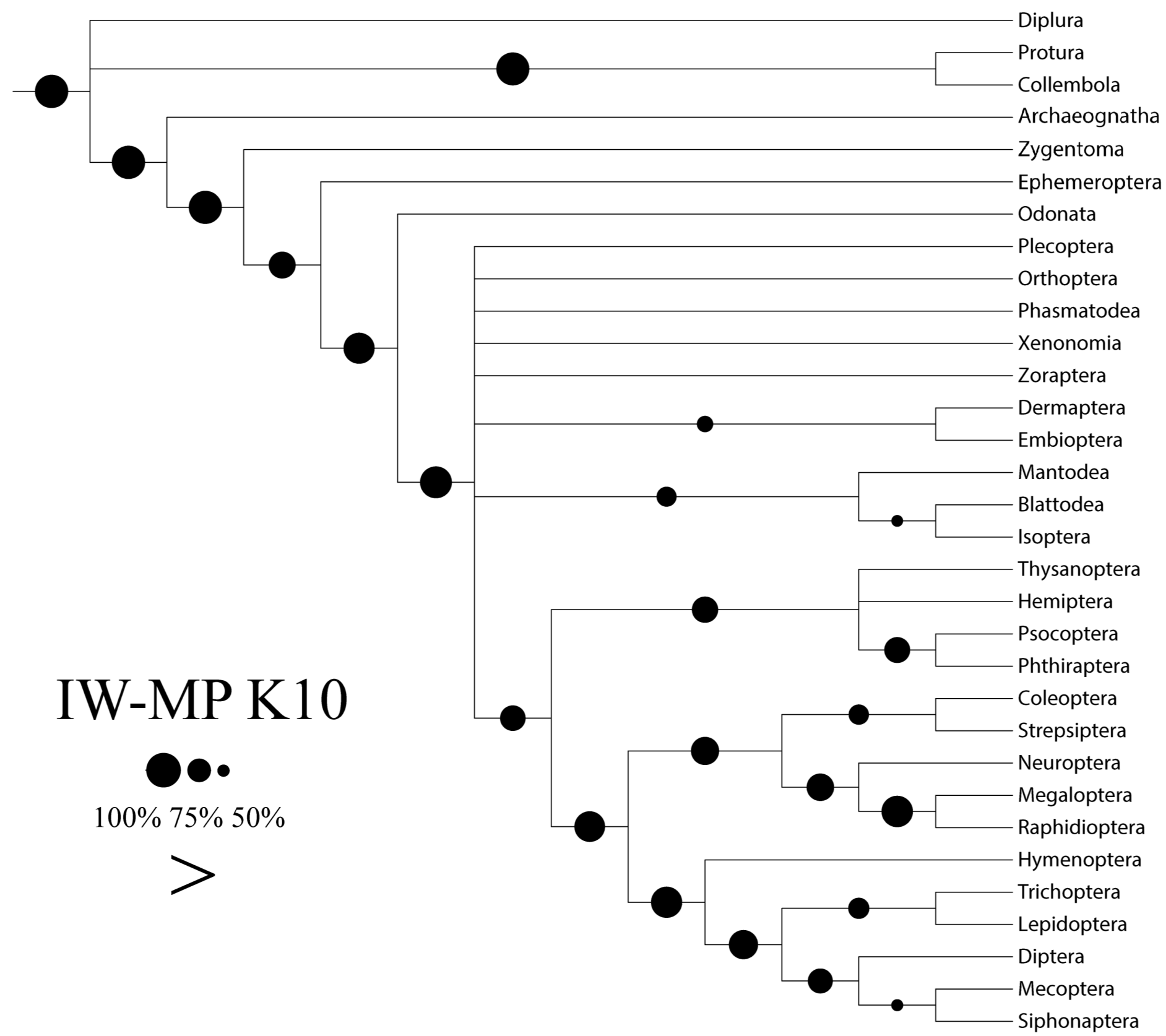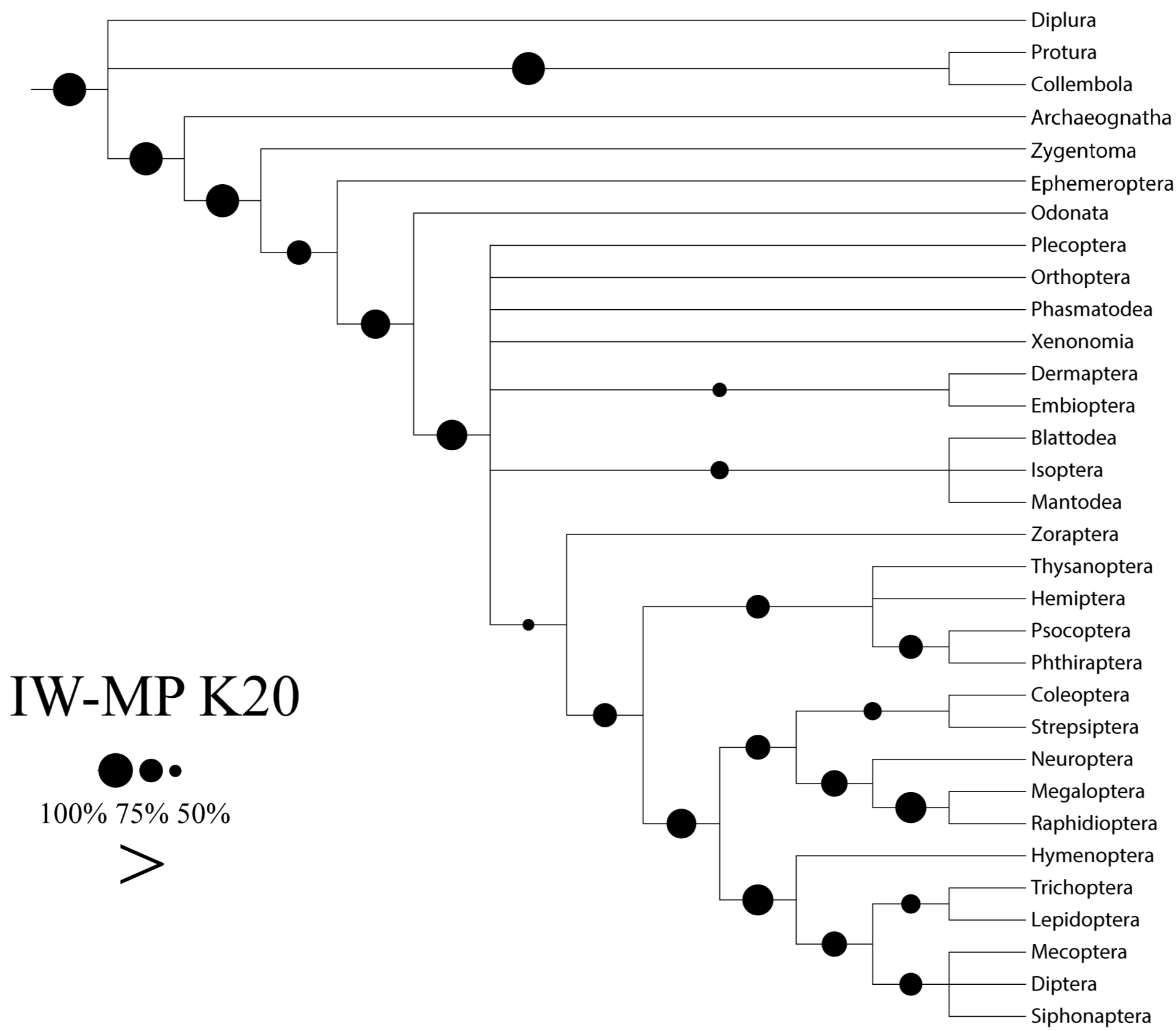

Supplement: Supplemental Information 9 [file peerj-12-16706-s009.pdf]

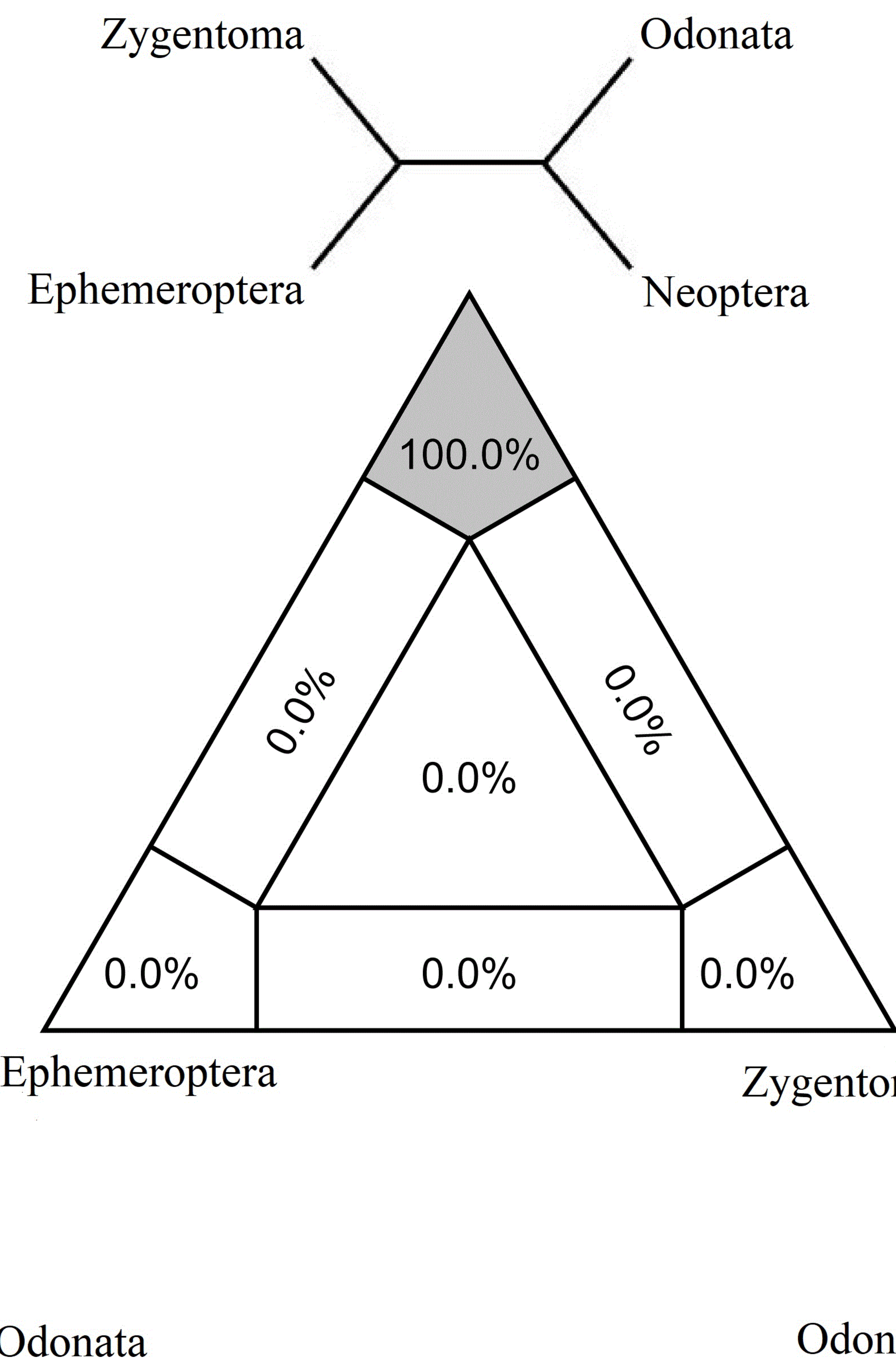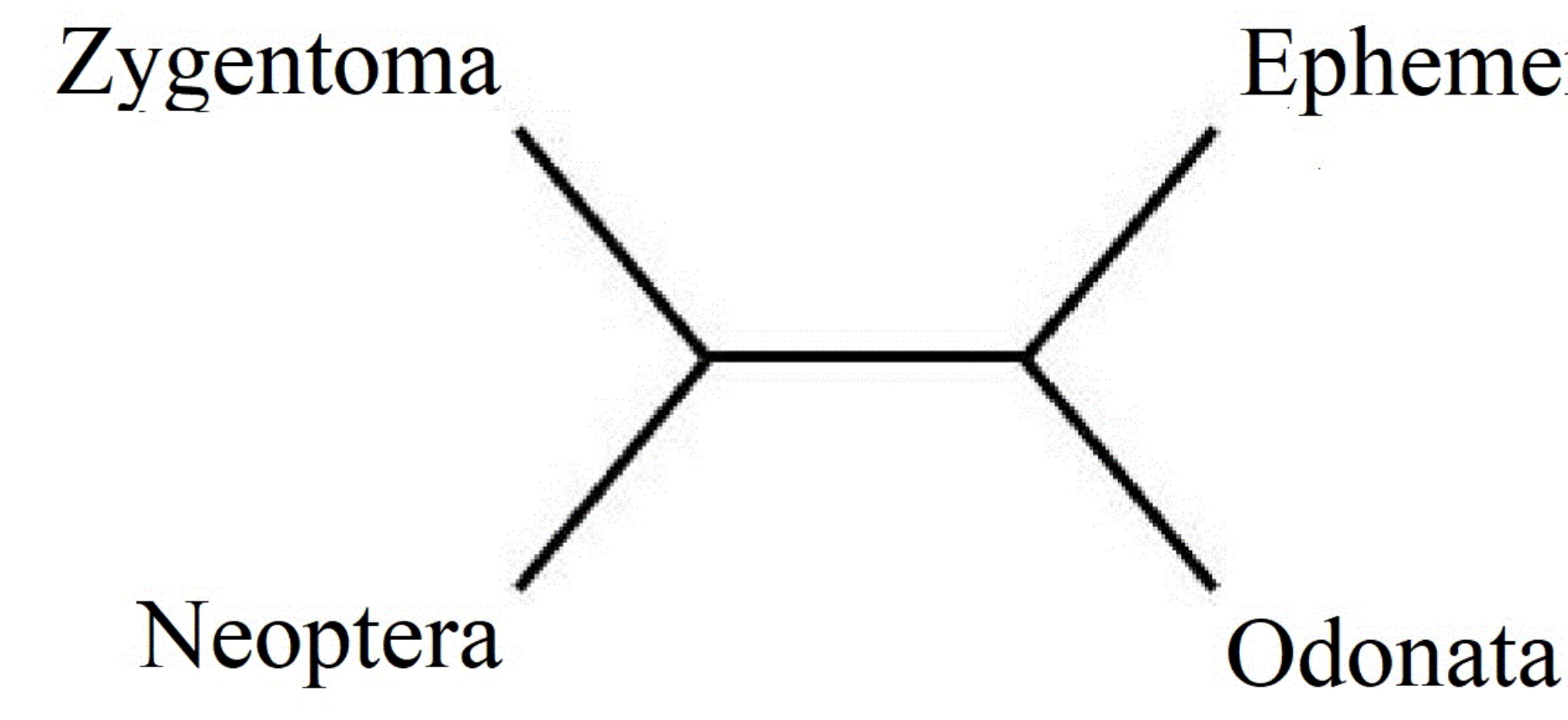

Reference (Misof *et al.*, 2014)

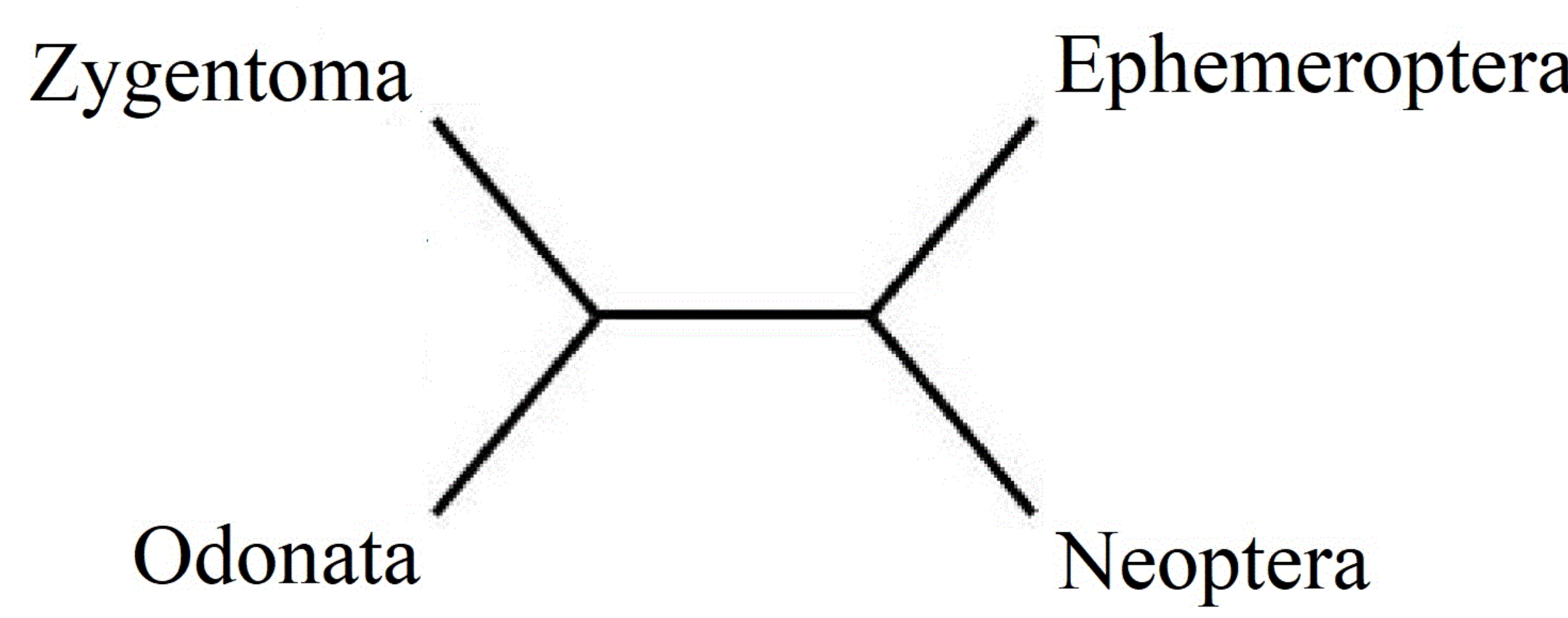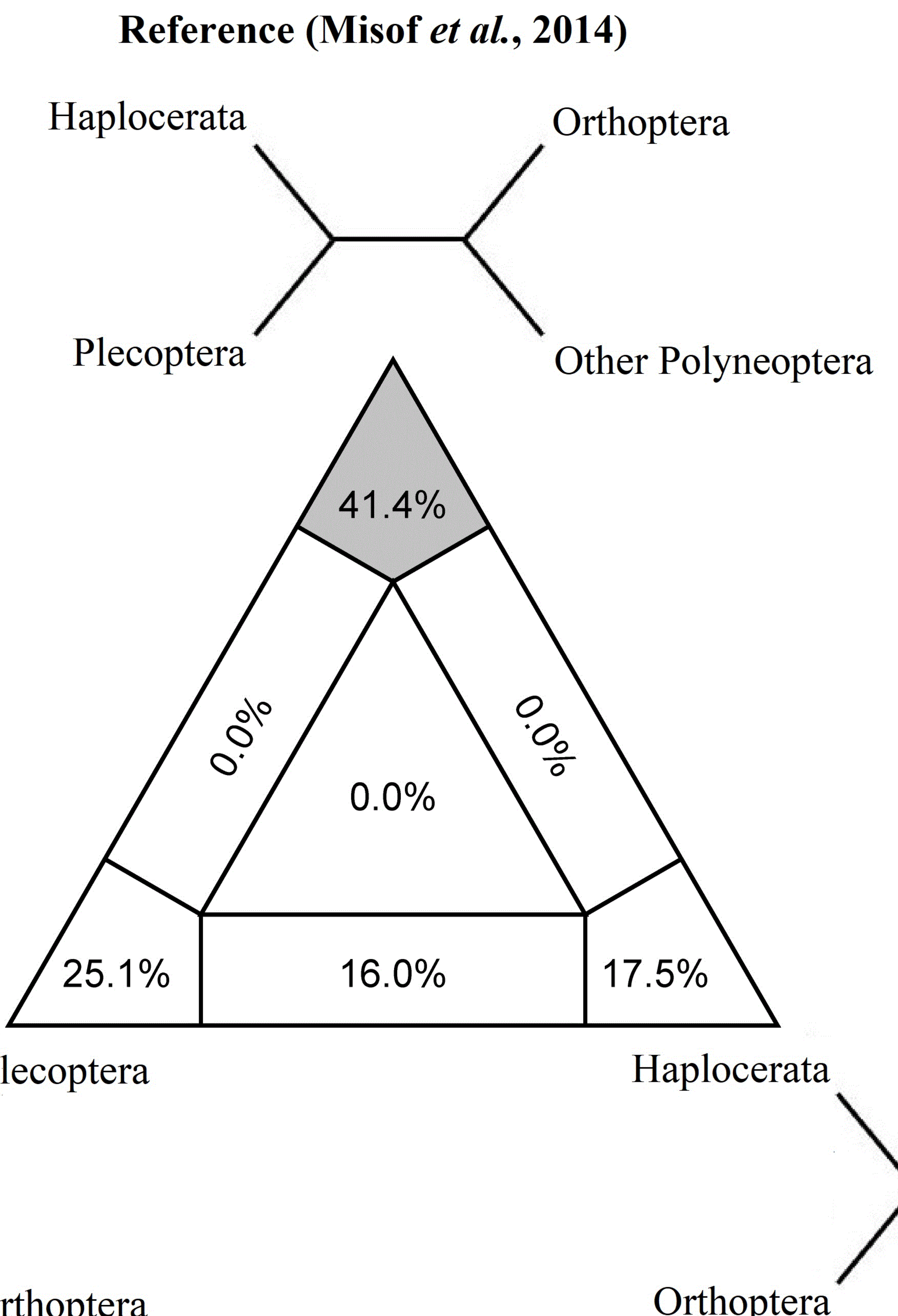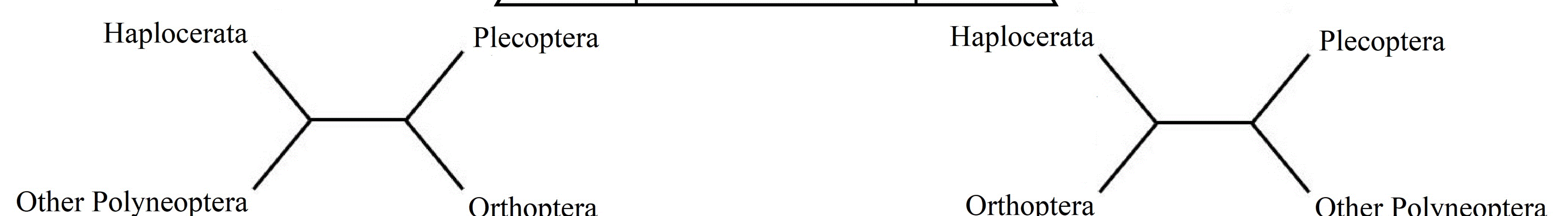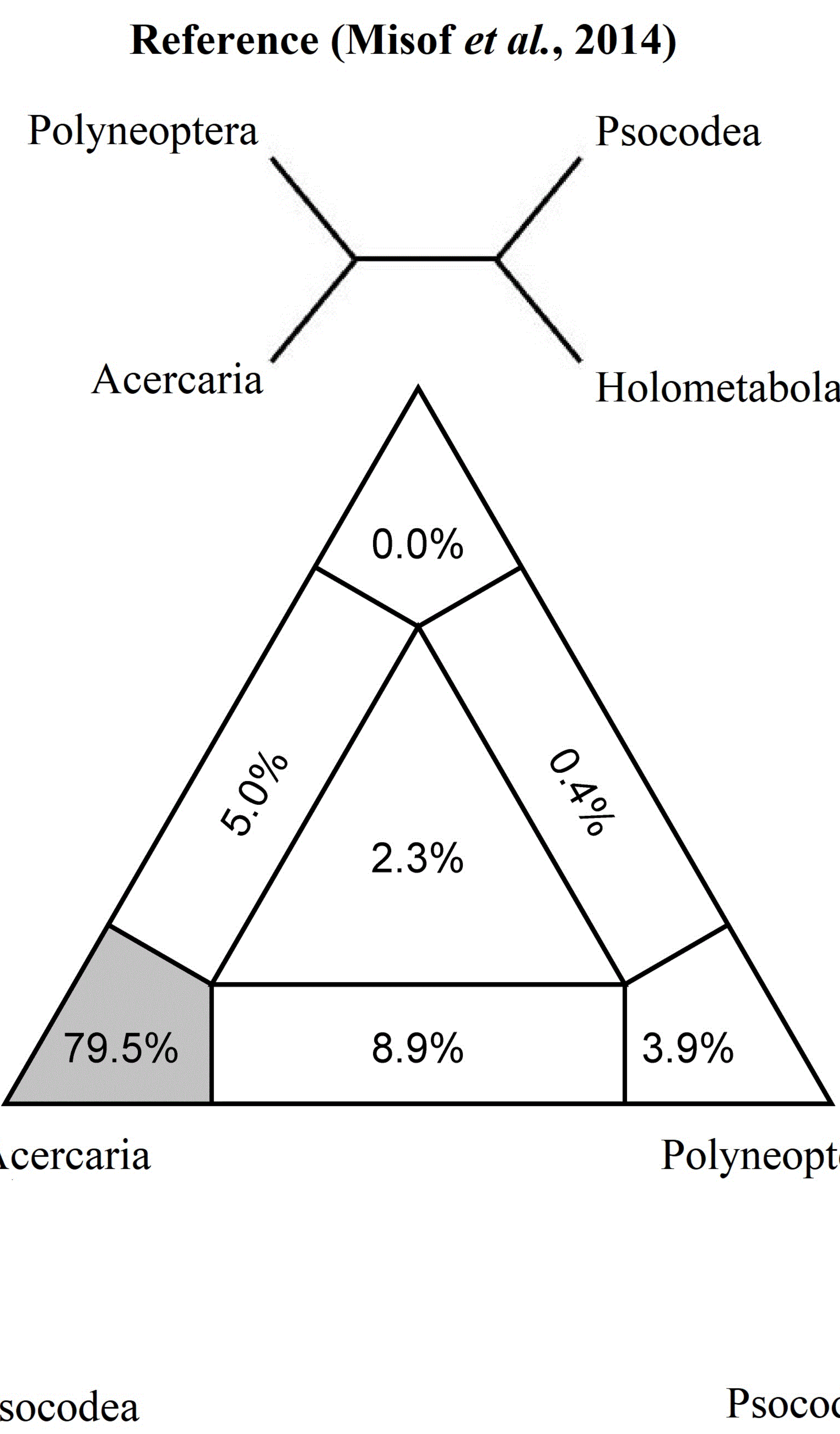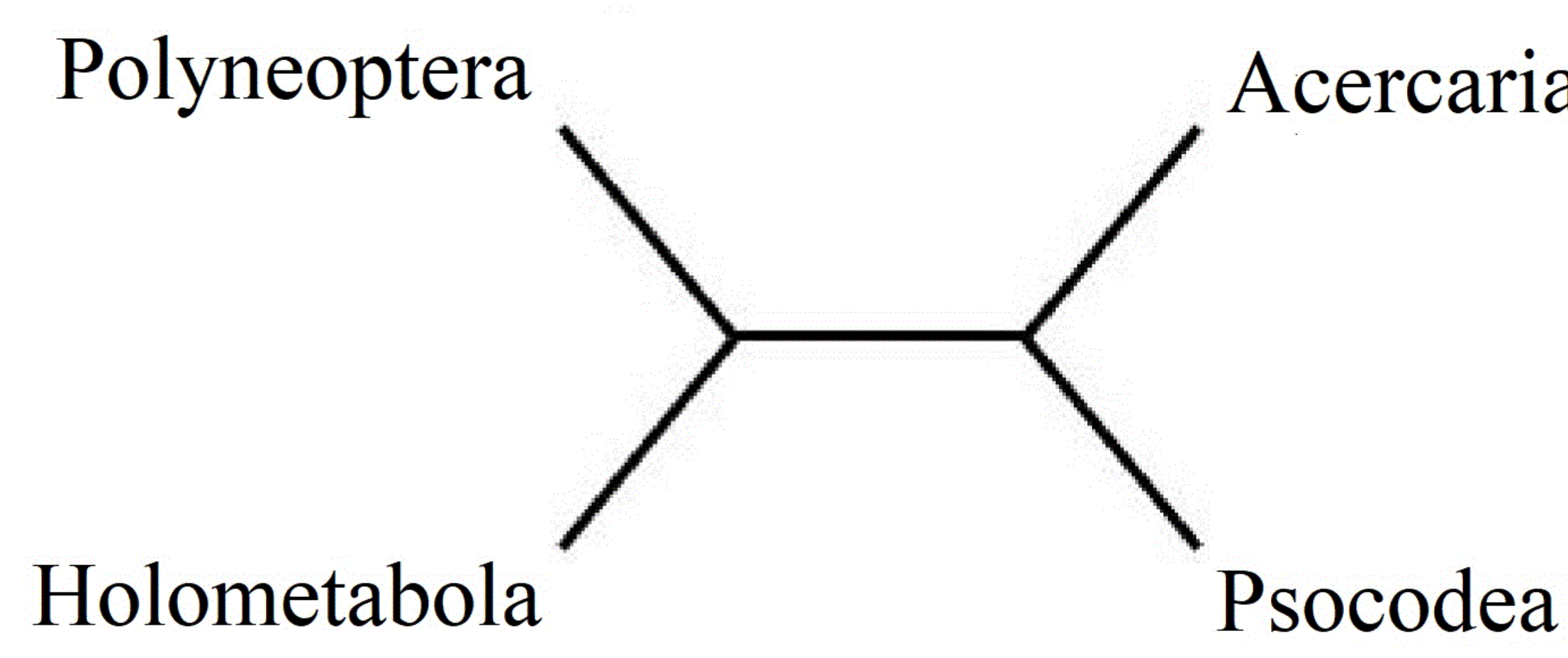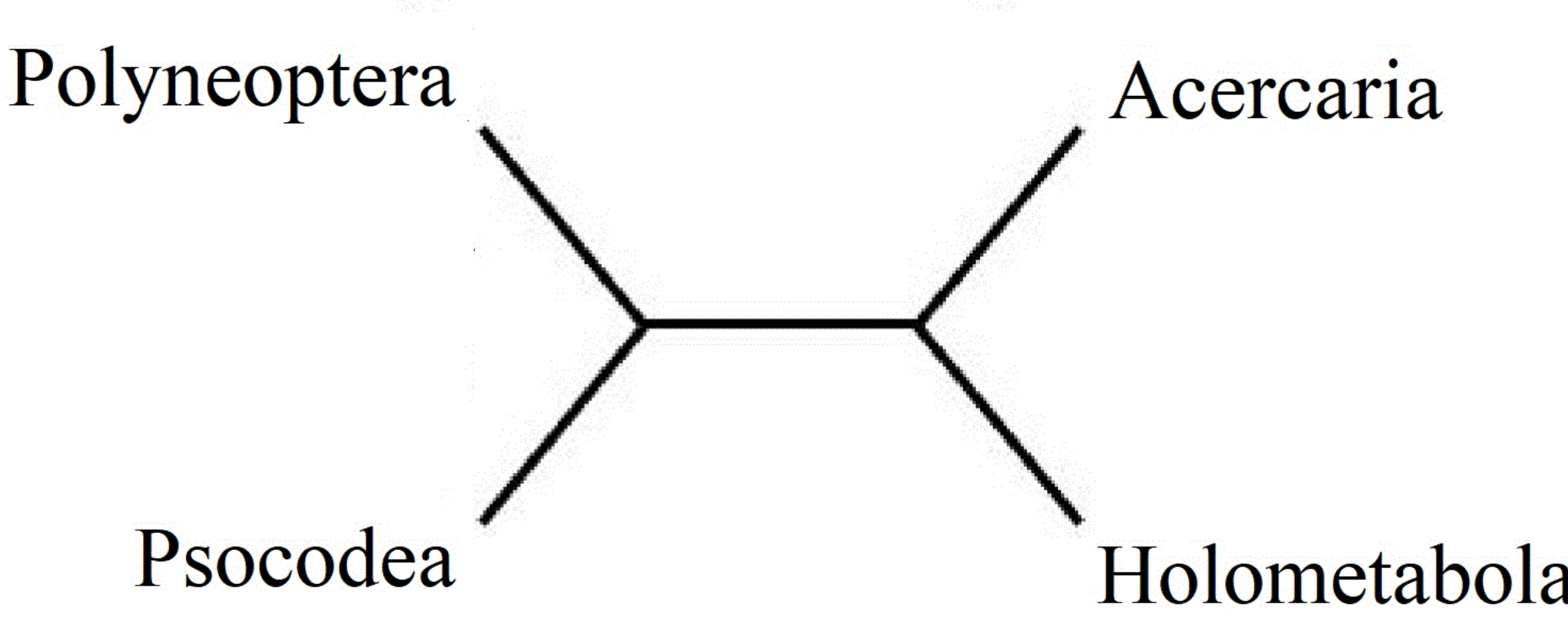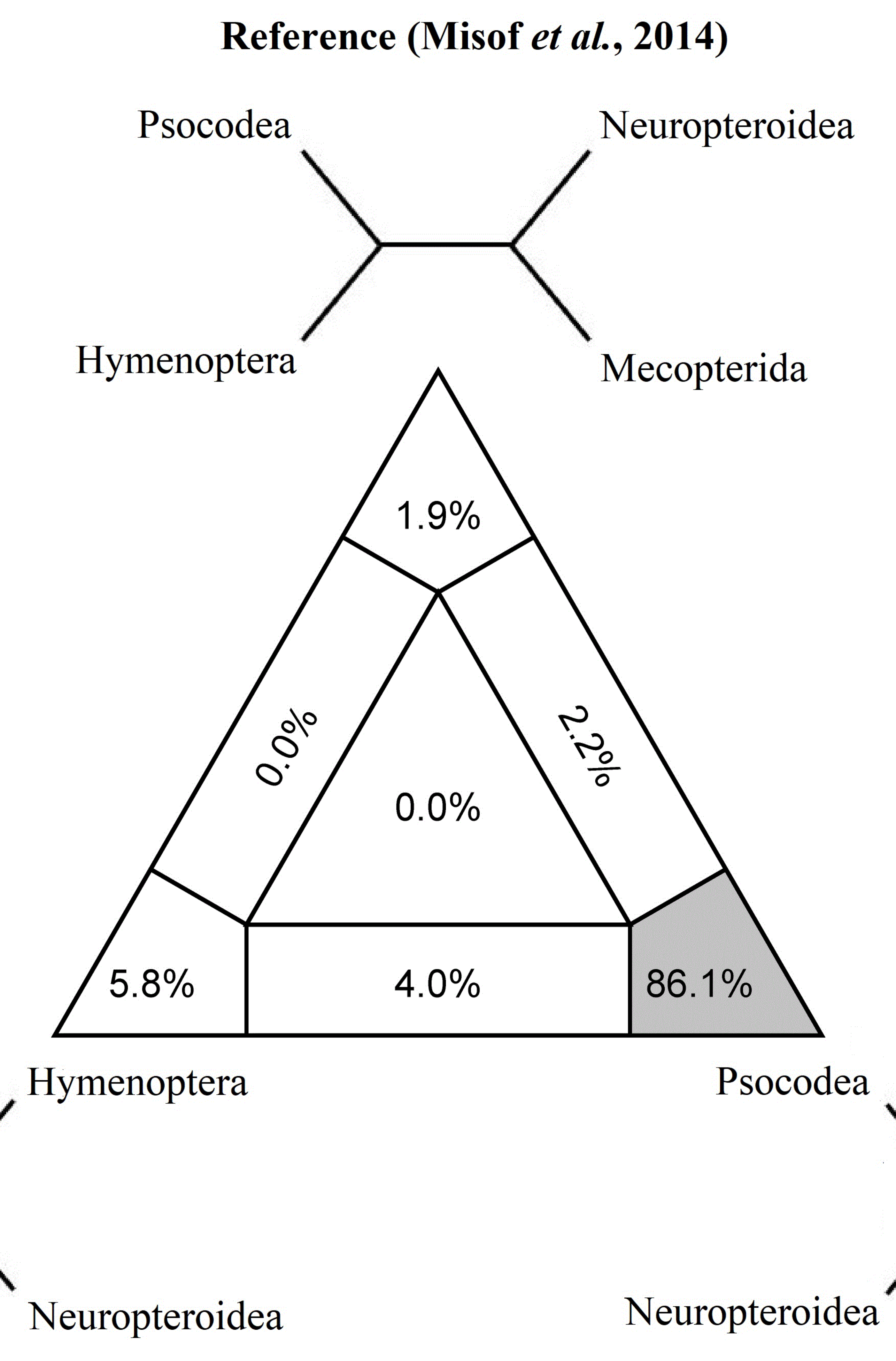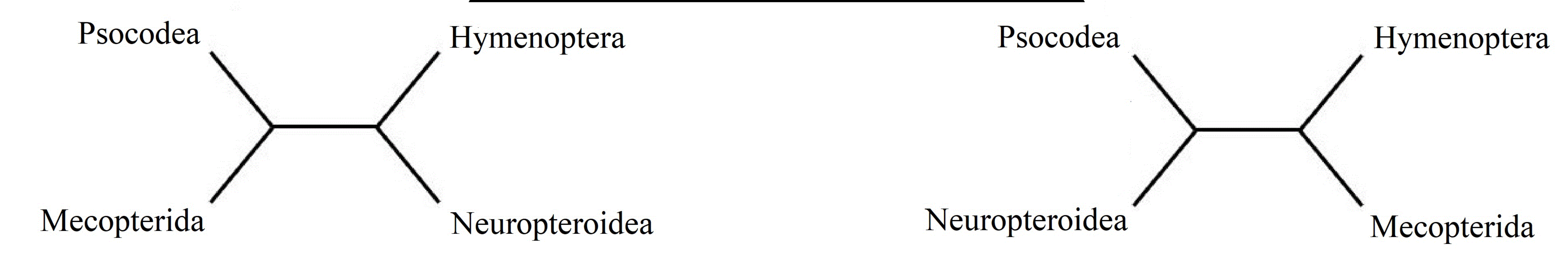

Reference (Misof *et al.*, 2014)

Supplement: Supplemental Information 11 — 2D simplex plot likelihood mapping analysis [file peerj-12-16706-s011.pdf]

Resolution (1-CFI)

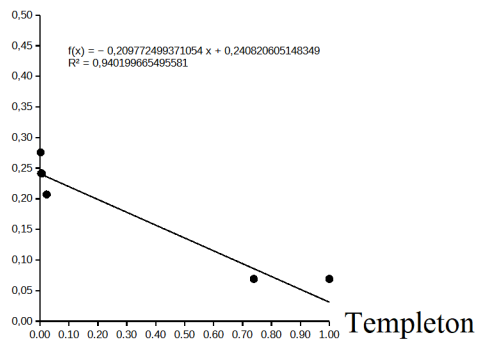

Resolution (1-CFI)

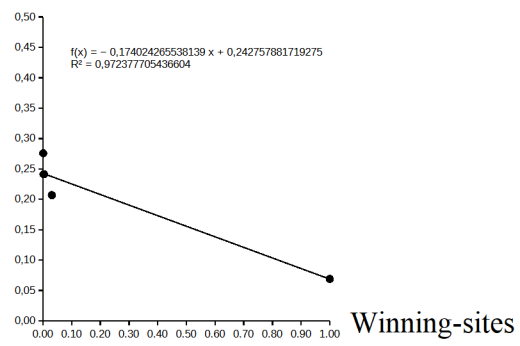

Resolution (1-CFI)

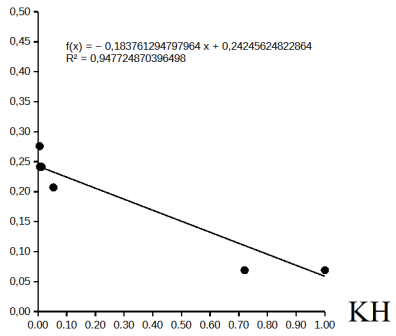

Resolution (1-CFI)

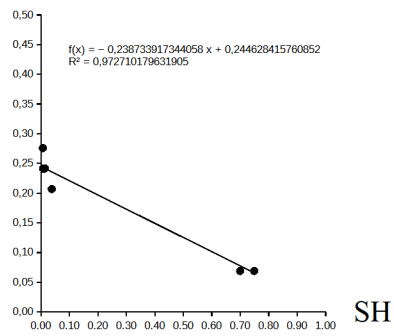

Resolution (1-CFI)

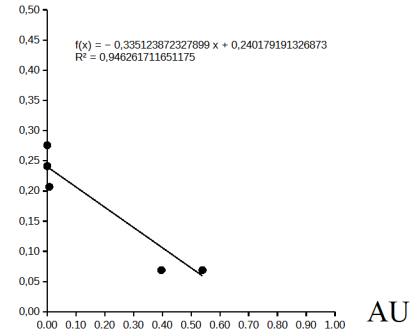

Supplement: Supplemental Information 12 [file peerj-12-16706-s012.pdf]
