# Supplementary material for: Performance of tree-building methods using a morphological dataset and a well-supported Hexapoda phylogeny
Source: PeerJ. 2024 Jan 8;12:e16706. doi: 10.7717/peerj.16706 (PMC10782957; doi:10.7717/peerj.16706)
Supplement: Supplemental Information 10 [file peerj-12-16706-s010.pdf]

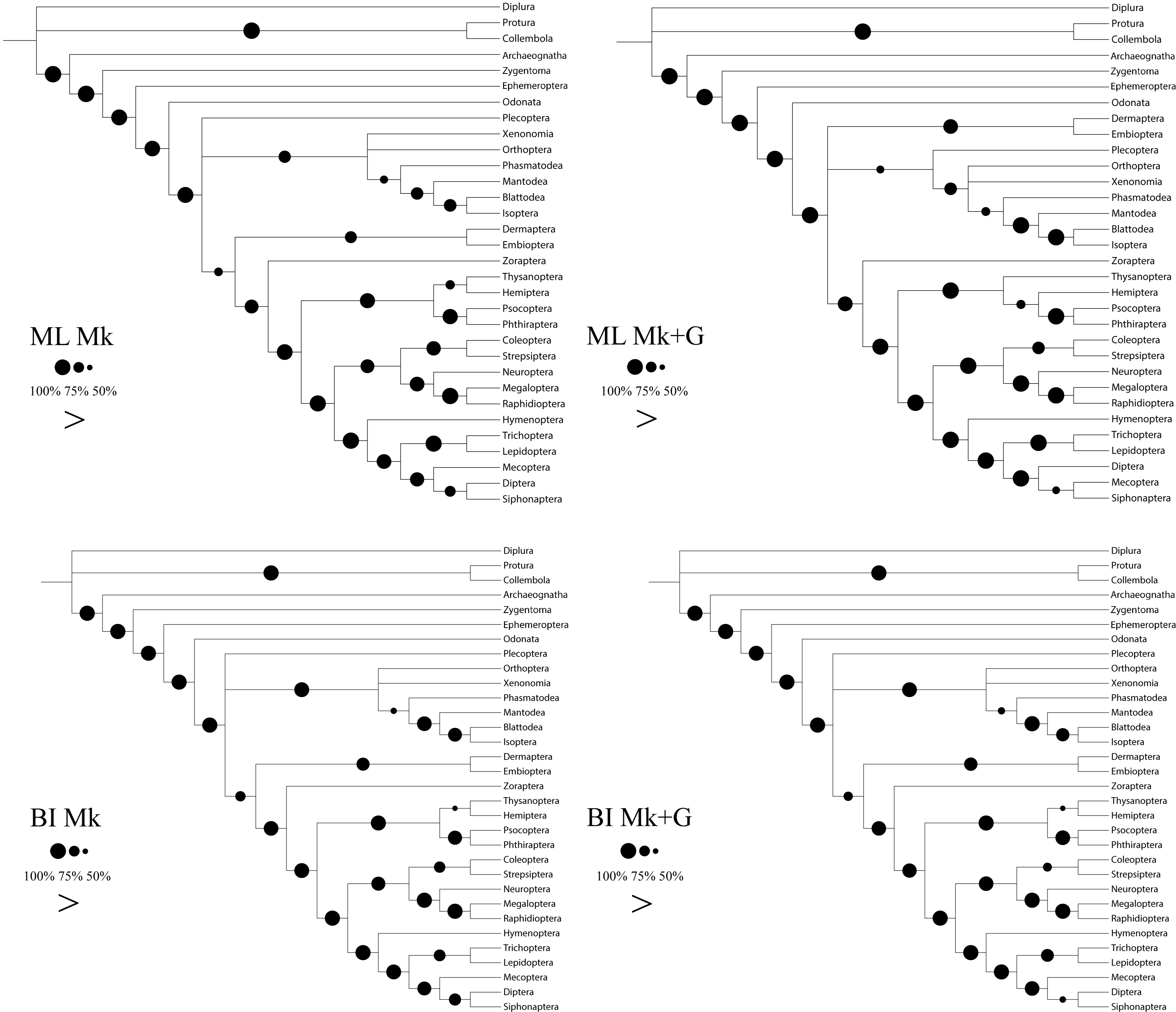
Diplura
Protura
Collembola
Archaeognatha
Zygentoma
Ephemeroptera
Odonata
Plecoptera
Xenonomia
Orthoptera
Phasmatodea
Mantodea
Blattodea
Isoptera
Dermaptera
Embioptera
Zoraptera
Thysanoptera
Hemiptera
Psocoptera
Phthiraptera
Coleoptera
Strepsiptera
Neuroptera
Megaloptera
Raphidioptera
Hymenoptera
Trichoptera
Lepidoptera
Mecoptera
Diptera
Siphonaptera
ML Mk
100% 75% 50%
>
Diplura
Protura
Collembola
Archaeognatha
Zygentoma
Ephemeroptera
Odonata
Dermaptera
Embioptera
Plecoptera
Orthoptera
Xenonomia
Phasmatodea
Mantodea
Blattodea
Isoptera
Zoraptera
Thysanoptera
Hemiptera
Psocoptera
Phthiraptera
Coleoptera
Strepsiptera
Neuroptera
Megaloptera
Raphidioptera
Hymenoptera
Trichoptera
Lepidoptera
Diptera
Mecoptera
Siphonaptera
ML Mk+G
100% 75% 50%
>
Diplura
Protura
Collembola
Archaeognatha
Zygentoma
Ephemeroptera
Odonata
Plecoptera
Orthoptera
Xenonomia
Phasmatodea
Mantodea
Blattodea
Isoptera
Dermaptera
Embioptera
Zoraptera
Thysanoptera
Hemiptera
Psocoptera
Phthiraptera
Coleoptera
Strepsiptera
Neuroptera
Megaloptera
Raphidioptera
Hymenoptera
Trichoptera
Lepidoptera
Mecoptera
Diptera
Siphonaptera
BI Mk
100% 75% 50%
>
Diplura
Protura
Collembola
Archaeognatha
Zygentoma
Ephemeroptera
Odonata
Plecoptera
Orthoptera
Xenonomia
Phasmatodea
Mantodea
Blattodea
Isoptera
Dermaptera
Embioptera
Zoraptera
Thysanoptera
Hemiptera
Psocoptera
Phthiraptera
Coleoptera
Strepsiptera
Neuroptera
Megaloptera
Raphidioptera
Hymenoptera
Trichoptera
Lepidoptera
Mecoptera
Diptera
Siphonaptera
BI Mk+G
100% 75% 50%
>
